# Supplementary material for: Red List assessment of amphibian species of Ecuador: A multidimensional approach for their conservation
Source: PLoS One. 2021 May 6;16(5):e0251027. doi: 10.1371/journal.pone.0251027 (PMC8101765; doi:10.1371/journal.pone.0251027)
Supplement: S1 Fig — (DOCX) [file pone.0251027.s010.docx]

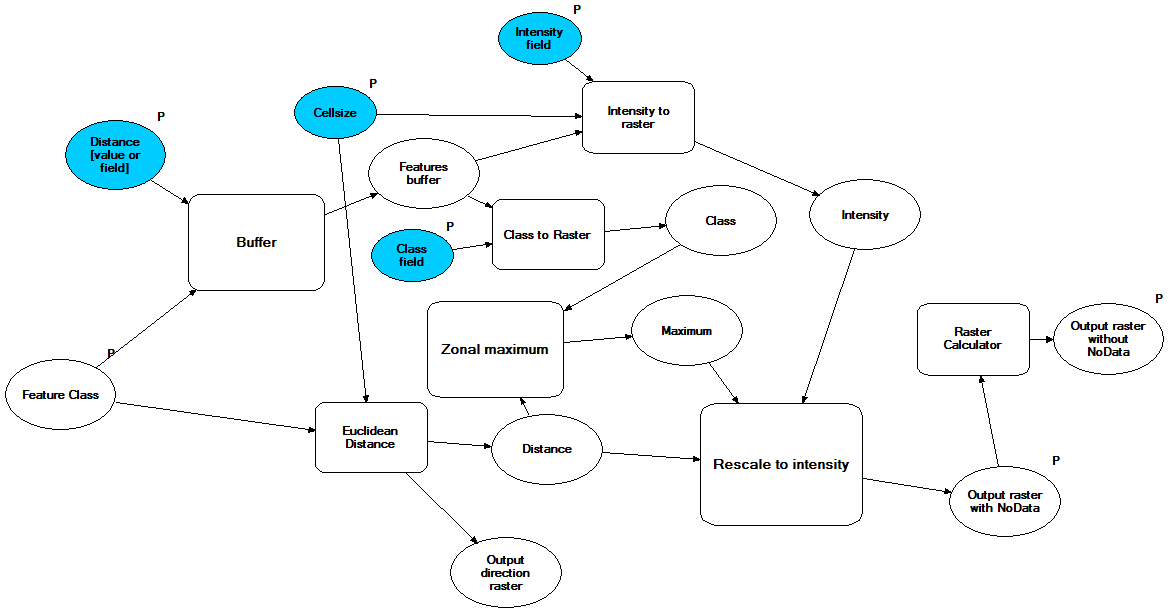


**S1 Fig** Automated procedure was designed using the *ModelBuilder* tool in ArcMap v.10 to perform the iterative threat model and its analysis.
