# Supplementary material for: Red List assessment of amphibian species of Ecuador: A multidimensional approach for their conservation
Source: PLoS One. 2021 May 6;16(5):e0251027. doi: 10.1371/journal.pone.0251027 (PMC8101765; doi:10.1371/journal.pone.0251027)
Supplement: S2 Fig — https://drive.google.com/file/d/1wkdx8DgDwKhVEyElDhc23wmEiknFw4DE/view?usp=sharing. (DOCX) [file pone.0251027.s011.docx]

**S2 Fig.** The threat model for Ecuadorian amphibians, raster image (.tiff / 427 Mb).

<https://drive.google.com/file/d/1wkdx8DgDwKhVEyElDhc23wmEiknFw4DE/view?usp=sharing>
