# Supplementary figures and images for: Red List assessment of amphibian species of Ecuador: A multidimensional approach for their conservation
Source: PLoS One. 2021 May 6;16(5):e0251027. doi: 10.1371/journal.pone.0251027 (PMC8101765; doi:10.1371/journal.pone.0251027)

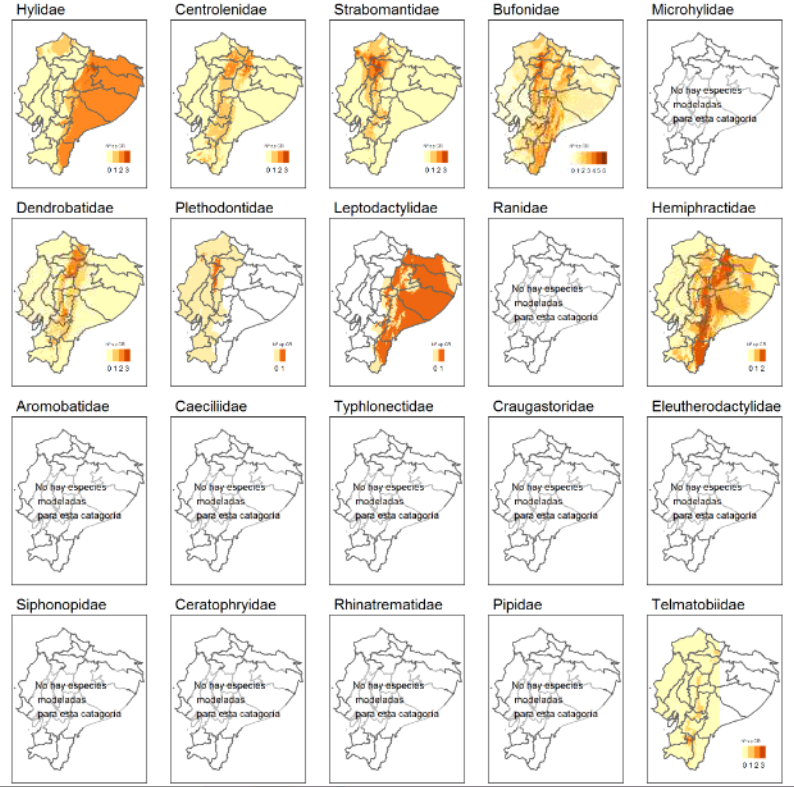


**S3 Fig.** Cumulative richness models of taxa qualified as Critically endangered by family.

Supplement: S3 Fig — (DOCX) [file pone.0251027.s012.docx]

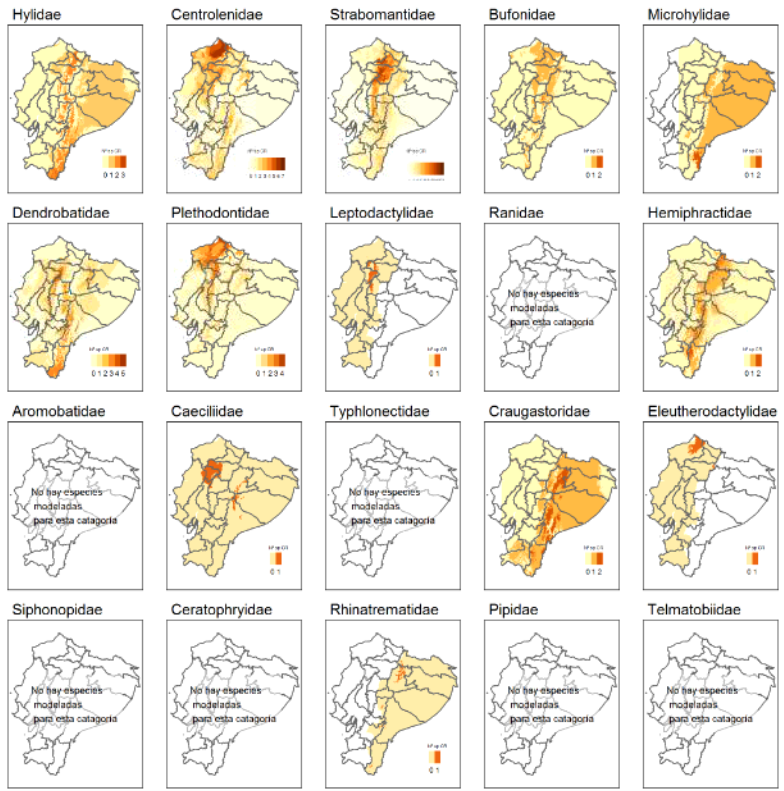


**S4 Fig** Cumulativerichness models of taxa qualified as Endangered by family.

Supplement: S4 Fig — (DOCX) [file pone.0251027.s013.docx]

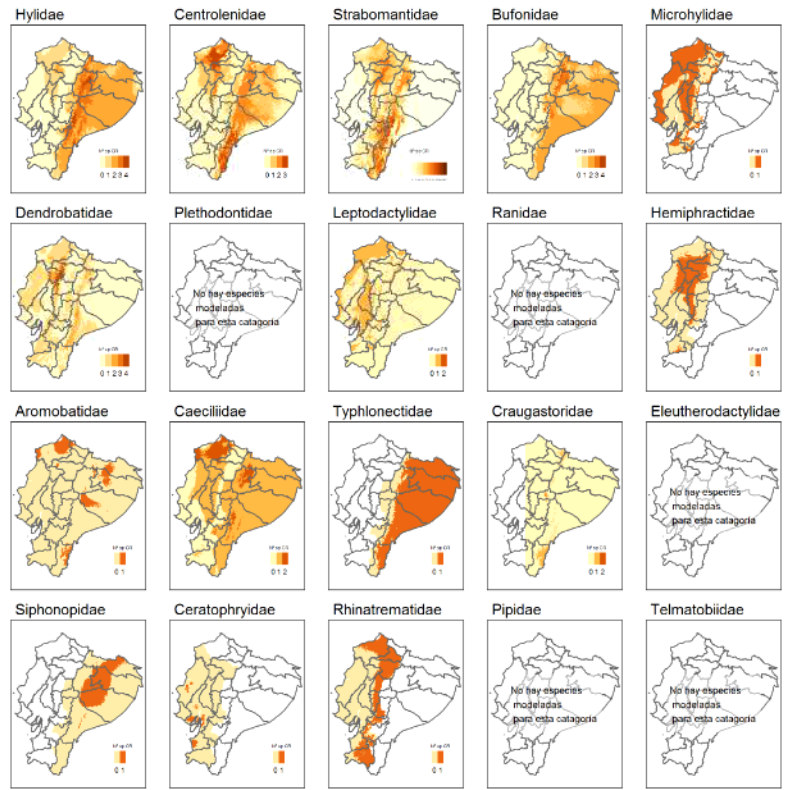


**S5 Fig** Cumulativerichness models of taxa qualified as Vulnerable by family.

Supplement: S5 Fig — (DOCX) [file pone.0251027.s014.docx]
