# Supplementary material for: Red List assessment of amphibian species of Ecuador: A multidimensional approach for their conservation
Source: PLoS One. 2021 May 6;16(5):e0251027. doi: 10.1371/journal.pone.0251027 (PMC8101765; doi:10.1371/journal.pone.0251027)
Supplement: Attachment — Submitted filename: PONE-D-20-34320_reviewer_luismarin.pdf [file pone.0251027.s015.pdf]

PLOS ONE

Red List assessment for amphibian species of Ecuador: a multidimensional approach for their conservation

--Manuscript Draft--

**Manuscript Number:**	PONE-D-20-34320
**Article Type:**	Research Article
**Full Title:**	Red List assessment for amphibian species of Ecuador: a multidimensional approach for their conservation
**Short Title:**	Red List assessment for Ecuadorian amphibians
**Corresponding Author:**	H. Mauricio Ortega-Andrade<br>Universidad Regional Amazónica IKIAM: Universidad Regional Amazonica IKIAM<br>Tena, Amazonia ECUADOR
**Keywords:**	Conservation; ecological niche modeling; threat modeling; risk categories
**Abstract:**	Ecuador is one of the most biodiverse countries in the world, but faces severe pressures and threats to its natural ecosystems. Numerous species have declined and require to be objectively evaluated and quantified, as a step towards the development of conservation strategies. Herein, we present an updated Red List Assessment for amphibian species of Ecuador, with one of the most detailed and complete coverages for any Ecuadorian taxonomic group to date. Based on standardized methodologies that integrate taxonomic work, spatial analyses, and ecological niche modeling, we assessed the extinction risk and identified the main threats for all Ecuadorian native amphibians (635 species), using the IUCN Red List Categories and Criteria. Our evaluation reveals that 57% (363 species) are categorized as Threatened, 12% (78 species) as Near Threatened, 4% (26 species) as Data Deficient, and 27% (168 species) as Least Concern. Our assessment almost doubles the number of threatened species in comparison with previous evaluations. In addition to habitat loss, the expansion of the agricultural frontier and other anthropogenic threats (roads, human settlements, and mining/oil activities) amplify the incidence of other pressures as relevant predictors of ecological integrity. Potential synergic effects with climate change and emergent diseases (apparently responsible for the sudden declines), has a particular importance amongst the threats sustained by Ecuadorian amphibians. Additional. Most threatened species are distributed in montane forests and paramo habitats of the Andes, with nearly 10% of them occurring outside the National System of Protected Areas of the Ecuadorian government. Also, it is essential to place research efforts on little known species categorized as Data Deficient (DD), which may turn out to be endangered. Such integration will help in better management and conservation of amphibian species in countries of the Tropical Andes Biodiversity Hotspot, like Ecuador. This assessment was a key step to develop the National Action Plan for the Conservation of Ecuadorian amphibians.
**Order of Authors:**	H. Mauricio Ortega-Andrade
	Marina Rodes Blanco
	Diego Francisco Cisneros Heredia
	Nereida Guerra Arévalo
	Karima Gabriela López de Vargas-Machuca
	Juan C. Sánchez-Nivicela
	Diego Armijos-Ojeda
	Jose Francisco Cáceres Andrade
	Carolina Reyes Puig
	Amanda Belén Quezada Riera
	Paul Székely

Powered by Editorial Manager® and ProduXion Manager® from Aries Systems Corporation

	Octavio R. Rojas Soto
	Diana Székely
	Juan M. Guayasamin
	Fausto Rodrigo Siavichay Pesántez
	Luis Amador
	Raquel Betancourt
	Salomón M. Ramírez Jaramillo
	Bruno Timbe Borja
	Miguel Gómez Laporta
	Juan Fernando Webster Bernal
	Luis Alfredo Oyagata Cachimuel
	Daniel Chávez Jácome
	Valentina Posse
	Carlos Valle Piñuela
	Daniel Padilla Jiménez
	Juan Pablo Reyes Puig
	Andrea Terán Valdez
	Luis A. Coloma
	María Beatriz Pérez Lara
	Sofía Carvajal Endara
	Miguel Urgilés
	Mario H. Yánez Muñoz
**Additional Information:**	
**Question**	**Response**
**Financial Disclosure**<br><br>Enter a financial disclosure statement that describes the sources of funding for the work included in this submission. Review the submission guidelines for detailed requirements. View published research articles from *PLOS ONE* for specific examples.<br><br>This statement is required for submission and **will appear in the published article** if the submission is accepted. Please make sure it is accurate.	This work was supported by the project "Conservation of Ecuadorian Amphibians and access to genetic resources-PARG"; projects "On the quest of the golden fleece in Amazonia: The first herpetological DNA - barcoding expedition to unexplored areas on the Napo watershed, Ecuador", funded by the Secretaría Nacional de Ciencia y Tecnología del Ecuador (Senescyt-ENSAMBLE Grant #PIC-17-BENS-001) and The World Academy of Sciences (TWAS Grant #16-095) granted to HMOA; and the project Critical Ecosystem Partnership Fund (CEPF) grant CEPF-108984 "Amphibian Conservation in the Abra de Zamora Key Biodiversity Area of Ecuador" granted to DAO, PS and DS.

**Unfunded studies**<br>Enter: *The author(s) received no specific funding for this work.*<br><br>**Funded studies**<br>Enter a statement with the following details:<br>• Initials of the authors who received each award<br>• Grant numbers awarded to each author<br>• The full name of each funder<br>• URL of each funder website<br>• Did the sponsors or funders play any role in the study design, data collection and analysis, decision to publish, or preparation of the manuscript?<br>• **NO** - Include this sentence at the end of your statement: *The funders had no role in study design, data collection and analysis, decision to publish, or preparation of the manuscript.*<br>• **YES** - Specify the role(s) played.<br><br>* typeset	
**Competing Interests**<br><br>Use the instructions below to enter a competing interest statement for this submission. On behalf of all authors, disclose any competing interests that could be perceived to bias this work—acknowledging all financial support and any other relevant financial or non-financial competing interests.<br><br>This statement **will appear in the published article** if the submission is accepted. Please make sure it is accurate. View published research articles from *PLOS ONE* for specific examples.	The authors have declared that no competing interest exist

Powered by Editorial Manager® and ProduXion Manager® from Aries Systems Corporation

**NO authors have competing interests**<br><br>Enter: *The authors have declared that no competing interests exist.*<br><br>**Authors with competing interests**<br><br>Enter competing interest details beginning with this statement:<br><br>*I have read the journal's policy and the authors of this manuscript have the following competing interests: [insert competing interests here]*<br><br>* typeset	
**Ethics Statement**<br><br>Enter an ethics statement for this submission. This statement is required if the study involved:<br><br>• Human participants<br>• Human specimens or tissue<br>• Vertebrate animals or cephalopods<br>• Vertebrate embryos or tissues<br>• Field research<br><br>Write "N/A" if the submission does not require an ethics statement.<br><br>General guidance is provided below. Consult the submission guidelines for detailed instructions. **Make sure that all information entered here is included in the Methods section of the manuscript.**	N/A

**Format for specific study types**<br><br>**Human Subject Research (involving human participants and/or tissue)**<br>• Give the name of the institutional review board or ethics committee that approved the study<br>• Include the approval number and/or a statement indicating approval of this research<br>• Indicate the form of consent obtained (written/oral) or the reason that consent was not obtained (e.g. the data were analyzed anonymously)<br><br>**Animal Research (involving vertebrate animals, embryos or tissues)**<br>• Provide the name of the Institutional Animal Care and Use Committee (IACUC) or other relevant ethics board that reviewed the study protocol, and indicate whether they approved this research or granted a formal waiver of ethical approval<br>• Include an approval number if one was obtained<br>• If the study involved *non-human primates*, add *additional details* about animal welfare and steps taken to ameliorate suffering<br>• If anesthesia, euthanasia, or any kind of animal sacrifice is part of the study, include briefly which substances and/or methods were applied<br><br>**Field Research**<br><br>Include the following details if this study involves the collection of plant, animal, or other materials from a natural setting:<br>• Field permit number<br>• Name of the institution or relevant body that granted permission	
**Data Availability**<br><br>Authors are required to make all data underlying the findings described fully available, without restriction, and from the time of publication. PLOS allows rare exceptions to address legal and ethical concerns. See the PLOS Data Policy and FAQ for detailed information.	Yes - all data are fully available without restriction

A Data Availability Statement describing where the data can be found is required at submission. Your answers to this question constitute the Data Availability Statement and **will be published in the article**, if accepted.<br><br>**Important:** Stating 'data available on request from the author' is not sufficient. If your data are only available upon request, select 'No' for the first question and explain your exceptional situation in the text box.<br><br>Do the authors confirm that all data underlying the findings described in their manuscript are fully available without restriction?	
**Describe where the data may be found in full sentences. If you are copying our sample text, replace any instances of XXX with the appropriate details.**<br><br>• If the data are **held or will be held in a public repository**, include URLs, accession numbers or DOIs. If this information will only be available after acceptance, indicate this by ticking the box below. For example: *All XXX files are available from the XXX database (accession number(s) XXX, XXX.).*<br>• If the data are all contained **within the manuscript and/or Supporting Information files**, enter the following: *All relevant data are within the manuscript and its Supporting Information files.*<br>• If neither of these applies but you are able to provide **details of access elsewhere**, with or without limitations, please do so. For example:<br><br>*Data cannot be shared publicly because of [XXX]. Data are available from the XXX Institutional Data Access / Ethics Committee (contact via XXX) for researchers who meet the criteria for access to confidential data.*<br><br>*The data underlying the results presented in the study are available from (include the name of the third party*	All relevant data are within the manuscript and its Supporting Information files.

and contact information or URL).<br>• This text is appropriate if the data are owned by a third party and authors do not have permission to share the data.<br><br>* typeset	
Additional data availability information:	

Manuscript

Click here to access/download;Manuscript;30Oct2020_PaperRL_PlainText.

Red List assessment for amphibian species of Ecuador: a multidimensional approach for their conservation

H. Mauricio Ortega-Andrade[1,13], Marina Rodes Blanco[1], Diego F. Cisneros-Heredia[2], Nereida Guerra Arévalo[1], Karima Gabriela López de Vargas-Machuca[3], Juan C. Sánchez-Nivicela[4], Diego Armijos-Ojeda[5], José Francisco Cáceres Andrade[6], Carolina Reyes Puig[2], Amanda Belén Quezada Riera[7], Paul Székely[8], Octavio R. Rojas Soto[9], Diana Székely[8], Juan M. Guayasamin[10], Fausto Rodrigo Siavichay Pesántez[11], Luis Amador[12], Raquel Betancourt[13], Salomón M. Ramírez-Jaramillo[14], Bruno Timbe-Borja[8], Miguel Gómez Laporta[14], Juan Fernando Webster Bernal[15], Luis Alfredo Oyagata Cachimuel[15], Daniel Chávez Jácome[14], Valentina Posse[7], Carlos Valle-Piñuela[15], Daniel Padilla Jiménez[15], Juan Pablo Reyes Puig[16], Andrea Terán-Valdez[17], Luis A. Coloma[17], Ma. Beatriz Pérez Lara[13], Sofía Carvajal-Endara[17], Miguel Urgilés[13] and Mario H. Yánez Muñoz[13]

[1] Grupo de Biogeografía y Ecología Espacial, Universidad Regional Amazónica Ikiam, Tena, Ecuador.

[2] Universidad San Francisco de Quito USFQ, Instituto de Diversidad Biológica Tropical iBIOTROP, Quito, Ecuador

[3] Universidad de La Laguna, Santa Cruz de Tenerife, España

[4] Universidad Nacional de Colombia, Bogotá, Colombia.

[5] Laboratorio de Ecología Tropical y Servicios Ecosistémicos (EcoSs-Lab), Departamento de Ciencias Biológicas, Universidad Técnica Particular de Loja, Loja 1101608, Ecuador.

[6] Parque Nacional Cajas ETAPA EP, Cuenca, Ecuador.

[7] Museo de Zoología de la Universidad del Azuay, Cuenca, Ecuador

[8] Laboratorio de Ecología Tropical y Servicios Ecosistémicos (EcoSs-Lab), Departamento de Ciencias Biológicas, Universidad Técnica Particular de Loja, Loja 1101608, Ecuador.

[9] Instituto de Ecología, A. C., Xalapa, México

[10] Laboratorio de Biología Evolutiva, Colegio de Ciencias Biológicas y Ambientales COCIBA, Instituto Biósfera USFQ, Universidad San Francisco de Quito, Quito, Ecuador.
[11] Centro de Conservación de Anfibios AMARU, Cuenca, Ecuador
[12] Instituto de Ciencias Ambientales y Evolutivas, Doctorado en Ciencias m. Ecología y Evolución, Universidad Austral de Chile, Valdivia, Chile.
[13] Instituto Nacional de Biodiversidad, Quito, Ecuador
[14] Independent researcher
[15] Ministerio del Ambiente y Agua, Quito, Ecuador
[16] Fundación Ecominga/Fundación Oscar Efrén Reyes, Baños, Ecuador.
[17] Centro Jambatu de Investigación y Conservación de Anfibios, Fundación Jambatu, Quito, Ecuador.

Author contribution

HMOA, MRB, NGA, DFCH, KGL and MHYM contributed in the logistics and organization of the workshops; HMOA, MRB, DFCH, NGA, KGL, JCSN, DAO, JCSN, CRP, ORRS, JMG, BTB and MHYM contributed in the concept and ideas on the manuscript design and analysis; HMOA, MRB, DFCH, NGA, KGL, JCSN, DAO, JCSN, CRP, ABQR, PS, DS, JMG, FRSP, LA, RB, SMRJ, BTB, MGL, JFWB, DCJ, VP, CVP, DPJ, JPRP, ATV, LAC, MBPL, SCE and MHYM contributed in data curation, codification and red list assessment criteria; HMOA, MRB, DFCH, NGA, KGL and MHYM analyze the data, generated models, designed figures and tables, and wrote the draft version of the manuscript; all authors reviewed, commented, and approved the final version of the manuscript; all authors declared not conflict of interest.

Abstract

Ecuador is one of the most biodiverse countries in the world, but faces severe pressures and threats to its natural ecosystems. Numerous species have declined and require to be objectively evaluated and quantified, as a step towards the development of conservation strategies. Herein, we present an updated Red List Assessment for amphibian species of Ecuador, with one of the most detailed and complete coverages for any Ecuadorian taxonomic group to date. Based on standardized methodologies that integrate taxonomic work, spatial analyses, and ecological niche modeling, we assessed the extinction risk and identified the main threats for all Ecuadorian native amphibians (635 species), using the IUCN Red List Categories and Criteria. Our evaluation reveals that 57% (363 species) are categorized as Threatened, 12% (78 species) as Near Threatened, 4% (26 species) as Data Deficient, and 27% (168 species) as Least Concern. Our assessment almost doubles the number of threatened species in comparison with previous evaluations. In addition to habitat loss, the expansion of the agricultural frontier and other anthropogenic threats (roads, human settlements, and mining/oil activities) amplify the incidence of other pressures as relevant predictors of ecological integrity. Potential synergic effects with climate change and emergent diseases (apparently responsible for the sudden declines), has a particular importance amongst the threats sustained by Ecuadorian amphibians. Additional. Most threatened species are distributed in montane forests and paramo habitats of the Andes, with nearly 10% of them occurring outside the National System of Protected Areas of the Ecuadorian government. Also, it is essential to place research efforts on little known species categorized as Data Deficient (DD), which may turn out to be endangered. Such integration will help

in better management and conservation of amphibian species in countries of the Tropical Andes Biodiversity Hotspot, like Ecuador. This assessment was a key step to develop the National Action Plan for the Conservation of Ecuadorian amphibians.

Keywords

Conservation, ecological niche modeling, threat modeling, risk categories.

Resumen

Ecuador es uno de los países con mayor biodiversidad del mundo, pero enfrenta severas presiones y amenazas a sus ecosistemas naturales. Numerosas especies han disminuido y requieren ser evaluadas y cuantificadas objetivamente, como un paso hacia el desarrollo de estrategias de conservación. A continuación, presentamos la Evaluación de la Lista Roja actualizada para especies de anfibios de Ecuador, basado en una de la más detallada y completa revisión hasta la fecha. Con base en metodologías estandarizadas que integran análisis taxonómicos, espaciales y modelado de nichos ecológicos, evaluamos el riesgo de extinción e identificamos las principales amenazas para todos los anfibios nativos ecuatorianos (635 especies), utilizando las Categorías y Criterios de la Lista Roja de la UICN. La evaluación revela que el 57% (363 especies) están categorizadas como Amenazadas, el 12% (78 especies) como Casi Amenazadas, el 4% (26 especies) como Datos Insuficientes y el 27% (168 especies) como Preocupación Menor. Nuestra evaluación casi duplica el número de especies amenazadas en comparación con evaluaciones anteriores. Además de la pérdida de hábitat, la expansión de la frontera agrícola y otras amenazas antropogénicas (carreteras, asentamientos humanos y actividades mineras /

petroleras) amplifican la incidencia de otras presiones como predictores relevantes de la integridad ecológica. Los potenciales efectos sinérgicos con el cambio climático y las enfermedades emergentes (aparentemente responsables de los descensos repentinos), tiene una importancia particular entre las amenazas que sufren los anfibios ecuatorianos. Adicional. La mayoría de las especies amenazadas se distribuyen en los bosques montanos y los hábitats de páramo de los Andes, y casi el 10% de ellas se encuentran fuera del Sistema Nacional de Áreas Protegidas del gobierno ecuatoriano. Además, es fundamental centrar los esfuerzos de investigación en especies poco conocidas categorizadas como Datos Insuficientes (DD), que pueden resultar en peligro de extinción. Tal integración ayudará a un mejor manejo y conservación de las especies de anfibios en países del Hotspot de Biodiversidad de los Andes Tropicales, como Ecuador. Esta evaluación fue un paso clave para desarrollar el Plan de Acción Nacional para la Conservación de los Anfibios Ecuatorianos.

Palabras clave

Categorías de riesgo, conservación, modelo de amenaza, modelos de nicho ecológico.

Introduction

One of the main aims of conservation biology is to assess, understand, and mitigate threats to biodiversity. The International Union for Conservation of Nature's (IUCN) Red List of Threatened Species is a powerful tool that allows not only to estimate species extinction risks, but also to prioritize conservation efforts [1]. The Red List Assessment is widely used by experts on several groups of plants and animals worldwide, as it applies standardized methods to assess threats and extinction risk, based on relevant quantitative and qualitative criteria [1-4].

Amphibians are one of the most diverse vertebrate groups in the Neotropical region [5]. In addition to presenting an extraordinary richness specific to each ecosystem, they are one of the most threatened taxa [6]. Their ectothermy makes them particularly vulnerable to environmental changes, mainly related to temperature and humidity, but also to infectious diseases [7-9]. Therefore, habitat loss, climate change, and diseases represent important threats to their populations [7,10-12].

Ecuador is one of the countries with the highest number of amphibian species [13-16]. Ecuadorian amphibians are considered among the most threatened in South America, due to increased rates of habitat loss and deforestation, mainly by cattle raising, mining, oil exploitation, and expansion of agricultural frontier [17-22]. Moreover, some historically conspicuous genera (harlequin frogs [*Atelopus* spp.], marsupial frogs [*Gastrotheca* spp.], and Andean water frogs [*Telmatobius* spp.]) have suffered dramatic populations declines or extinctions [9,23-25], that seem to be related to the fungal panzootic *Batrachochytrium dendrobatidis* [9], although other factors, such as climate change may be also be related [11].

Based on the data gathered by the IUCN Red List Assessment, amphibians are the most threatened vertebrates globally, and the proportion of threatened species increases more rapidly than both birds and mammals [26-28]. By March 2020, from an estimated 8126 amphibian species, 6824 were evaluated (84% of the known species), and 2202 (32% of the evaluated species) were considered threatened [species assessed as Critically Endangered (CR), Endangered (EN), or Vulnerable (VU)]. However, globally, the proportion of threatened amphibian species would increase in a range between 41 and 53% if we considered that several Data Deficient (DD) species are likely to be in fact threatened with extinction [29,30].

In 2004, the Global Amphibian Assessment (GAA) published by the IUCN, Conservation International and NatureServe included, for the first time, the categorization for amphibian species of Ecuador; this was subsequently updated in 2006 and 2008 (www.iucn-amphibians.org). As a result of this process 447 amphibian species were evaluated, from which 165 (37%) were found to be threatened or extinct [4]. In 2011, an updated assessment was published for Ecuadorian species [28], with 465 evaluated species, 142 (30.5%) of which were found to be threatened (CR, EN or VU) and nearly 29% classified as DD.

Since 2015, the Ecuadorian Ministry of Environment and Water (MAAE) has been leading the project “Conservation of Ecuadorian amphibian biodiversity and sustainable use of genetic resources”. One of the main components of the project is focused on understanding the conservation status of the amphibians of Ecuador. Thus, the goals of our study are to: a) evaluate and update the extinction risk status of Ecuadorian amphibians, b) analyze spatial patterns of threatened species related to endemism, protected areas, and ecological regions

in Ecuador, and c) suggest actions towards a robust and objective methodology to evaluate species conservation status.

Materials and methods

Amphibian database compilation

In order to gather the distribution data for Ecuadorian amphibians, we compiled occurrence records along the complete distributional range per species from: Global Biodiversity Information Facility (GBIF; https://www.gbif.org), iNaturalist (https://www.iNaturalist.org), VertNet (https://www.vetnet.org), Batrachia (https://www.batrachia.com), Anfibios del Ecuador [15], Museo de Zoología de la Pontificia Universidad Católica del Ecuador (QCAZ; https://bioweb.bio), as well as from national databases shared by the Instituto Nacional de Biodiversidad (INABIO), Museo de Zoología de la Universidad Técnica Particular de Loja (MUTPL), Museo de Zoología de la Universidad del Azuay (MZUA), Museo de Zoología de la Universidad Tecnológica Indoamérica (MZUTI), Museo de Zoología de la Universidad San Francisco de Quito (ZSFQ), Centro Jambatu (CJ), Proyecto Conservación de Anfibios y Recursos Genéticos del Ministerio de Ambiente del Ecuador (MAE-PARG), and records/photographs of specimens collected in the field and shared by the authors in the Red List Assessment workshops (S1 Table). The final dataset included data up to 31th October 2020 (Fig 1).

We followed the nomenclature proposed by Grant et al. [31] for Dendrobatidae, Guayasamin et al. [32] for Centrolenidae, Castroviejo-Fisher et al. [33] for Hemiphractidae, Hedges et al. [34] for Strabomantidae; all other taxa groups follow AmphibiaWEB [13] and The Amphibian Species of the World [16]. Records

were error-checked and improved through a taxonomic assessment on specimens in scientific collections, validation of records based on biogeographic distribution, phylogenetic and taxonomic analyses published elsewhere [35-43], a systematic literature review and by taxonomic discussions in eight workshops (2017 to 2020) that were held with 33 expert herpetologists from all over the country, including the authors of this paper. Workshop participants were distributed on boards according to taxonomic families and geographic regions. Spatialized data per species was revised in QGIS 3.4.14, along with geospatial data as watersheds, digital elevation model, and base maps, in order to assess for data consistency [44]. As a double-check, information of elevation was extracted for every data point and represented in boxplots to find outliers and other possible mistakes. Problematic occurrence data, either at georeferenced or taxonomic level, were removed from the dataset. Taxonomic experts validated the data and highlighted errors or inaccuracies during workshops. Records with incorrect georeferenced data were fixed using the Google Satellite layer in QGIS, only when the collectors verified the exact location. This process aimed to obtain a clean and debugged database that met appropriate standards for ecological niche modeling [45-47], biogeographic analyses [48,49], and Red List Assessment [13,17,22], in accordance with Darwin Core guidelines (https://dwc.tdwg.org/).

Environmental Data

Climate variables for current and future scenarios were downloaded from the WorldClim2 database [50] (http://www.worldclim.org). We obtained 15 climatic variables at a 30 seconds (~1 km^2) spatial resolution; we excluded the four layers that combine precipitation and temperature information into the same layer due

to spatial anomalies [51]. To characterize future climate conditions, we used data for two IPCC representative concentration pathways emissions scenarios (RCP 4.5 and 8.5) from the Hadley Global Environment Model 2 - Earth System (HadGEM2-ES) global circulation model (GCM) [50]. Future RCP 4.5 scenarios assume relative slow income growth, increasing human population and modest improvements in technology and energy intensity, leading to a higher demand for energy and increasing greenhouse gas emissions in the long-term considering an absence of climate change mitigation policies, whereas the RCP 8.5 scenario represents a higher predicted greenhouse gas emissions [52].

Data Analysis and Ecological Modeling

Species were divided into two groups: 1) those that could be modeled, and 2) those that could not be modeled due to low number of occurrence points (fewer than 5 localities) occurrence points situated in closely-located pixels, or models not statistically significant by AUC thresholds. For the first group, we implemented a modeling process with MaxENT [53]. The characteristics of the model (creation, calibration, selection, and evaluation) were carried out in ***kuenm*** R package [54]. As a first step, the Jackknife procedure and the correlation statistics (-0.8 to 0.8 in *Pearson r* values) were used to assess the importance of the variables in a first run with all values by default.

Once the climate variables were selected, we obtained candidate models with different parameters (seven multiplication regulators - 0.1, 0.4, 0.7, 1, 2, 3, 4 - and seven feature classes - linear (*l*), quadratic (*q*), product (*p*), and all the combinations *lq, lp, pq, lqp*-). The maximum number of background points was 10,000. We randomly selected 70% of the data for training and used the remaining 30% for testing. A total of 500 runs were set for model building. The

best model was selected according to the criteria of omission rate < 10% and delta AIC > 2.

An important step in ecological niche modeling is to define a calibration region, the accessible area (“M”, hereinafter) for species [45,47,55]. In this study, we delimited “M" using the biogeographic provinces for the Neotropics [56], watersheds, and a digital elevation model to find the physical barriers that determine the accessibility area of each amphibian species. We found similar distribution patterns among several species, reiterating the same physical barriers (i.e. the Andes, basins, mountain ranges, etc.). For these reasons, some generic “M" were constructed for the different regions (i.e. highlands, coast, and Amazon), and these were assigned to each of the species.

In the case of taxa that lacked enough data points for ecological modeling, the Area of Occupancy (AOO) was calculated [57] in R software (https://www.r-project.org/), using a 2 x 2 km grid created in QGIS 3.4.14 and extracting and counting the number of cells occupied by the species.

Cumulative Species Richness Model

The cumulative species richness models (CSRM) were performed adding up the results of the Maxent binary models (suitability area) and Area of Occupancy (AOO) for each of the families and conservation categories. The results are shown using the ***tmap*** package [58] in R software (https://www.r-project.org/).

Endemic species were determined based on the categories proposed by Ron et al. [15]. We used a Kruskal-Wallis test and a Wilcoxon test for paired samples to compare groups of endemic/ non-endemic taxa and conservation threat categories related to altitude ranges.

Threat Model

To have a better understanding of the potential impacts of human activities on the distribution of Ecuadorian amphibians, we followed standardized criteria to define risk elements and potential threats, based on expert supervision for hierarchical classification by IUCN-CMP (International Union for Conservation of Nature - Conservation Measures Partnership) [3,57,59]. Overall, eight major threats with 34 subcategories were used to develop a threat or Environmental Risk Surface (ERS) model (Table 1).

We used a standard lexicon for classifications of threats [59]. These elements were spatially mapped (ArcMap v.10) as points, polygons, and lines, and then converted to raster files to calculate Euclidean distances of each threat. The Influence Distance (meters) was assigned to each subcategory based on buffer areas with a respective decay function, giving values according to the intensity of anthropogenic and natural threats. To reduce subjectivity by decision-making bias, regarding the ascription of Intensity to each risk element, we applied a Multi-Criteria Decision Making (MCDM) through Analytic Hierarchy Process (AHP) on the analysis (S3 Table). Once the inputs were obtained, the process was automatized using *ModelBuilder* from ArcMap, with an iterative process per subcategory (S1 Fig.). Finally, the outputs were overlapped with a raster calculator to develop an ERS, which considers a weighted overlay of amphibians-specific threats in Ecuador, with a resolution of 30 m x 30 m.

Red List Assessment

The conservation status of amphibian species in Ecuador was assessed following the protocols, standards, criteria, and subcriteria proposed by the IUCN [4]**.** The dataset was compiled in a geospatial database used to assess the distribution

and threats in a series of workshops promoted by the working group led by the authors. Data by species were analyzed mainly by number of records (N), percentage of records in Ecuador (%), area of occurrence (AOO, km^2), suitability area reconstructed by niche modeling (km^2), environmental contractions [60,61] in future scenarios (% reduction relative to current ecological model), and values higher than 0.5 (in the third quartile) of the threat model.

All statistics (43 in total) used to apply criteria and subcriteria to assess the conservation status of a given species are detailed in S2 Table. Additional data related to population size or decline of the number of mature individuals were documented from literature or data from the authors provided in the workshops. As additional support for the evaluation, we used basic maps for National System of Protected Areas (SNAP - Sistema Nacional de Áreas Protegidas), Forestal Heritage, Protected forests and vegetation, Conservation Areas, Ramsar wetlands, Land Use and forested areas (until 2018) and Natural Regions of Ecuador, downloaded in vector format from national servers [28,62-64]. We calculated the threatened representativeness in a taxonomic group (TR): the number of threatened taxa / total number of taxa per family X 100. Comparative assessment of threatened taxa regarding the last National Red List follows Ron et al. [28].

Results

Red List Assessment

A total of 126 databases belonging to various institutions and on-line resources were used to consolidate the dataset for the Ecuadorian amphibians (S1 Table). The final dataset included 37,328 records, from which 29,189 were located in

Ecuador, of a total of 635 taxa (plus *Rana catesbeiana*, as an invasive species), which represent 100% of the species currently reported for Ecuador (Fig 2). GBIF, QCAZ, and INABIO were the data providers with the most representative collections included in the current Red List evaluation (Table 2).

Overall, the IUCN Red List assessment resulted in the assignment of a threatened category (CR, EN, VU) for 57% of the Ecuadorian amphibian species, while 12% were considered as Near Threatened (NT), 4% as DD, and 27% as Least Concern (LC) (Fig 2, Table 3). Eighty-five taxa were considered as Critically Endangered CR (13.4%), including taxa from the genera *Atelopus* (24 spp.), *Hyloxalus* (9 spp.), and *Pristimantis* (12 spp.); 147 taxa (23.1%) were considered Endangered (EN), and 131 (20.6%) qualified as Vulnerable (VU). Strabomantidae is the family with the highest number of threatened taxa (CR = 18 species, 3%, EN = 67 species, 11.1%; VU = 87 species, 14.5%, respectively). Strabomantidae (28.6%), Bufonidae (7%), and Centrolenidae (6.3%) harbor 42% of the total threatened species in Anura. An additional 78 taxa (12.3%) were evaluated as NT, and 168 as LC (26.4%). Finally, 26 taxa (4.1%) are considered as DD because the information was insufficient for a proper assessment of their conservation status (Fig 3, S3 Table). Regarding the taxa under threatened categories, 56.7% (341 species) of Anura, 72.7% (8 species) of Caudata, and 60.9% (9 species) of Gymnophiona qualified for one of these categories (Table 3). A total of 16 genera had all of their taxa considered as threatened [i.e. *Atelopus* (25 spp.), *Lynchius* (4 spp.), *Epicrionops* (3 spp.), *Telmatobius* (3 spp.), *Ctenophryne* (3 spp.), *Sachatamia* (3 spp.)]; seven genera had 70–90% of taxa as threatened [i.e. *Hyloxalus* (22 spp.), *Nymphargus* (15 spp.), *Gastrotheca* (14

spp.)]; 12 genera had 50-70% as threatened [i.e. *Pristimantis* (155 spp.), *Hyloscirtus* (13 spp.), *Caecilia* (7 spp.)] (Fig 3, S5 Table).

A total of 287 species (45%) occurring in Ecuador are endemic. Four families (*Andinobates*, *Ectopoglossus*, *Paruwrobates* and *Telmatobius*) have all their endemic species as threatened; the families Bufonidae, Dendrobatidae, Strabomantidae have 70–90% of their endemic species categorized as threatened. 18 genera have all of their endemic taxa evaluated as threatened (*i.e. Atelopus, Lynchius, Niceforonia, Paruwrobates, Rhaebo, Telmatobius)* and 10 (*Caecilia, Chiasmocleis, Epipedobates, Espadarana, Gastrotheca, Hyloscirtus, Hyloxalus, Nymphargus, Osornophryne, Pristimantis)* have been identified with 70-90% of their endemic species as threatened (S5 Table). Our assessment incorporates 178 species that had never been evaluated; also, we present the conservation status for 127 species that were considered as DD in previous red list evaluations (S3 Table).

Major Threats

The ERS model is presented in S2 Fig This model reveals high-risk areas (red) mainly located in the vicinity of large and medium-sized cities: Guayaquil (Coast), Quito (Andes), and Lago Agrio (Amazon). The medium-to-high-risk areas (orange) are primarily placed on the eastern and western foothills of the Andes mountain range, northern Amazonia, and northern Coast, with high threats scattered on central Coast and Amazonia regions, nearby roads. Medium-risk areas (yellow) can be identified along the Andes, as well as in the center-southern part of the Coast. We noticed that the areas of low impact (green) are isolated,

related to protected areas, inaccessible forests, and mountain ranges located in northwestern Ecuador, Amazonian foothills of the Andes, and southern Amazonia (Fig 4).

Agriculture, transport, infrastructure (i.e. roads, oil pipelines, etc.), production areas (mining, oil camps, etc.) and deforestation are the most important threats for Ecuadorian amphibians, with 70-98% taxa associated to each of these categories (Fig 5, S7 Table). Near to 21-36% of assessed species will have a contraction in more than a half of the area that represents their ecological niches (loss of environmental conditions, RCP 45/85) in future scenarios. We documented the presence of *Rana catesbeiana*, an introduced species, in several locations mainly distributed in southern slopes of the Andes and coastal regions.

Biogeographical patterns

Cumulative species richness models (CSRM) by threatened category are shown in Figure 6 (models per species, genera, and families are detailed in Supplementary Material SM4). CSRM for threatened species generated a maximum value of 57 species overlapped per pixel. A high concentration of threatened taxa is related to the northern montane forests in both sides of the Andes, paramos, and valleys in the central Andes and eastern montane forest towards southern to Cutucú and Condor ranges and foothills of the Amazon basin (Fig 6).

CR taxa CSRM generated a maximum value of 12 species overlapped per pixel. A high concentration of taxa is located along both sides of the Andes, in northern

Ecuador near Cayambe Coca Ecological Reserve and Napo Sumaco-Galeras National Park, and the montane forest of southeastern Ecuador close to the Cutucú and Condor Mountain ranges. Models for EN taxa generated a maximum value of 28 species overlapped per pixel. The highest concentration of taxa was in the northwestern Andes, in areas west of Pichincha volcano, Mindo, Guayllabamba basin in Esmeraldas, Pichincha, Imbabura and Carchi provinces. Models for VU taxa generated a maximum value of 27 species overlapped per pixel. The higher concentration of VU taxa was located along with mountain forests and foothills in both sides of the Andes, in the Chocó region, in nearby areas of Napo Sumaco-Galeras National Park and southeastern Ecuador (Fig 6).

Locality records of threatened species reveal differential patterns of distribution depending on the family (Fig 7). For example, Bufonidae, Centrolenidae, Dendrobatidae, and Strabomantidae are related to the Andes and foothills. Telmatobiidae, which have all of their species categorized as CR, is restricted to southern Andes (Fig 7). Strabomantidae is the only family that presents CR taxa limited to the coastal region. On the other hand, Hylidae and Leptodactylidae have been recorded on both sides of the Andes, related to foothills and tropical forests. Threatened salamanders (Caudata, Plethodontidae) have been registered in northern Ecuador, towards foothills on both sides of the Andes, and tropical forests in the Chocó region (Fig 7).

Records of NT taxa are distributed on both sides of the Andes by Centrolenidae, Dendrobatidae, and Hylidae; while Hemiphractidae and Leptodactylidae are represented mainly in the Amazon basin and eastern slopes of the Andes. A

wider distribution of locality records in Ecuador (except the dry area in the coastal region) of NT taxa is identified for Strabomantidae. DD taxa are mostly located in the foothills and lowlands along the Amazon region, mainly for Bufonidae, Hylidae, Aromobatidae, and Centrolenidae; also, DD species in families Strabomontidae have been registered in the Andes (Fig 8).

The database had records from lowlands to highlands in Ecuador (min = 6 m, 1st Qu. = 821 m, median = 1694 m, mean = 1760 m, 3rd Qu. = 2728 m, max. = 5299 m). We report differences in the distribution of Red List categories and endemic taxa related with altitude [KW test (χ^2) = 591.58, d.f. = 5, p<2.2e-16]. Threatened species were distributed commonly in highlands, montane forests, and foothills of Andes i.e. CR (median = 2240 m, n = 1159), EN (median = 1862 m, n = 2096), VU (median = 1533 m, n = 3599), compared with NT taxa (Fig 7).

The highest number of species was essentially encountered in three natural regions: eastern montane (318 taxa), western montane (224 taxa), and the Amazon (208 taxa). The montane regions also harbored the highest proportions of threatened, 27% for each one, and DD of the total species in Ecuador. Regarding species richness in each region, the paramo had the highest proportion of threatened species (80%), followed by the western montane (74%), Andean shrub (69%), and western foothills (65%). (Fig 9, S6 Table).

The Vegetation and Protected Forests and the SNAP protected areas are the most important types of protected areas for threatened amphibian species, with an overall record of 203 (32%) and 196 species (31%), respectively (Fig 10, S6

Table). Sixty-five species (10%) are not included in any protected area, with 26 rated as CR, 25 as EN, and 14 as VU.

Discussion

The current conservation status of Ecuadorian amphibians

The conservation status of 635 (plus *R. catesbeiana*, as invasive species) amphibian species was assessed, which represents all the native species documented to date for Ecuador (S4 Table). Herein we report that 57% of the evaluated amphibian species are classified under some extinction risk using the IUCN Red List standards (13% CR, 23% EN, and 21% VU), with a further 12% falling into the NT category, and 4 % of DD taxa. Our data present a rather pessimistic situation of one of the most diverse countries in amphibian species in the World [16]. This is especially true as the data are correlated with the fact that Ecuador also boasts one of the highest deforestation rates [21,65], an immense pressure for mining development [66], and an important expected human population growth in the future [67].

Compared to the previous Ecuadorian Amphibian Red List [28], we add assessments for 174 species, and additionally provide a status evaluation for 127 species that were considered DD at that time (S3 Table). As a result of our study, the conservation status of 139 taxa has changed - 81 species have now been found to qualify in a higher Red List category, while 58 have been assigned to a lower extinction risk category. The differences are probably due to broader knowledge, including taxonomic revisions and species descriptions, but also to the different assessment procedures.

Amidst a general trend of loss of biodiversity, some amphibian taxa show a phylogenetic sensitivity to change, as they are considered at high risk of extinction in their entirety (i.e. genus *Atelopus*, *Telmatobius*, *Lynchius,* etc.), most likely as a result of their distinctive life-history traits [24,40,68-71]. Because they contribute uniquely to the functioning of their communities, the loss of such species is especially worrisome as it is expected to have a disproportionate impact on the stability of local ecosystems, beyond their taxonomic loss [11]. This is of particular importance since most of them are endemic species not only for Ecuador, but also for specific habitats [15].

Major Threats

We have generated a quantitative and objective ranking of threats for Ecuadorian amphibians, using clear and comprehensive rules [59]. A ranking of threats helps to identify and prioritize the conservation actions needed to mitigate them and allows results that are comparable and replicable [72]. Agriculture is of particular importance amongst the threats experimented by Ecuadorian amphibians. In Ecuador, the unsustainable use of forested lands and agriculture-related deforestation, even in areas where human population is low, are important threats making a priority the need for better strategies to improve the rural population management practices. Also, some anthropogenic threats (roads, human settlements, and oil activities) amplify the incidence of other pressures and, as shown in previous studies, are the most relevant predictors of ecological integrity [1,36,73].

The ecological characteristics and microhabitat preferences of the species can lead to deep variations in the susceptibility to certain drivers of extinction

amongst taxa [2]. In amphibians, species respond differently to disturbance [74], therefore a distinction should be made, and conservation measures to be adopted must be different along environmental gradients [75]. For example, we found a different distribution pattern in the case of threatened species, as well as endemic ones, both showing a higher density along an altitudinal gradient, with a peak toward montane forests and highlands (Fig 7). However, cases of amphibian species interaction with spatial patterns of human impacts are puzzling. An alarming trend is that the greatest density of endangered taxa occurs in montane and paramo ecosystems, regions that we would expect to be under a lesser anthropic impact. Further considerations on climate change and synergic effects with habitat loss and emergent diseases, like Chytridiomicosis, must be considered as major threats to Ecuadorian amphibians [11,69], especially to endemic species.

Future assessment efforts should include the presence of invasive species as another potential threat to Ecuadorian amphibians. Currently, there are few studies focused on determining the expansion of these species and their effect on native amphibian populations. The bullfrog (*R. catesbeiana)* has been reported in six Ecuadorian provinces [76]; rainbow trout (*Oncorhynchus mykiss*) and common trout (*Salmo trutta*) are present in Andean areas of the whole Ecuadorian highlands [77]. The threat that these species represent to amphibians could be significant, considering their predatory and expansionist biology, but also because it generally overlaps with other threats that affect the habitat of species listed at extinction risk. A special case is represented by the Galápagos Islands, which do not have any native amphibian species, but scattered records

of established tree-frog populations (*Scinax quinquefasciatus*) are reported in Santa Cruz and Isabela islands [15,78]. The effects of this species on the local ecosystems should be monitored in the future.

Protected areas and threatened species

An evaluation of existing protected areas overlapped with the endangered species distribution reveals that much work is still needed to ensure the long-term survival of amphibians. Since the existence of protected areas is considered the main hope for preserving threatened species from extinction [79], the fact that 10% (65 species) of the Ecuadorian threatened amphibian species occur uniquely outside protected areas is preoccupying and highlights the limitations of the current National Protected Areas Network.

Our study emphasizes several areas of that are home of a high number of threatened amphibian species and that are not protected (Fig 10). This is especially evident in three locations: the Chocó area (north of the "Los Ilinizas" ecological reserve and the Pichincha volcano), the area among Cayambe-Coca, Antisana, and Sumaco, and in the southern part of the country (south of Sangay National Park). By including these areas with high amphibian species diversity (Figs. 4, 6 and 10) in the national protected area network would maximize ecological representativeness and threatened species' coverage [80].

The dataset of distribution records reveals an important sampling effort bias, mostly related to roads or accessible areas (Fig 1). Large areas have been under-sampled, especially coastal areas, Andean paramo, and Amazonia. As a result, species categorized as DD are mostly located in the Amazon Region and on the eastern slopes of the Andes (Fig 8). In many cases, the remoteness of the areas

prevents access due to logistical difficulties [80]. Although for the same reasons, the anthropic impact should be lower, in the case of high-altitude Andean habitats, we notice an overlapped high density of threatened species (especially CR), emphasizing the importance of focused searches for healthy populations in these secluded regions.

In the case of coastal areas, the shortage of inventories is not caused by limited access, severe habitat loss, but rather of insufficient sampling. Although a lower amphibian diversity is likely, mainly because of extreme climatic factors that restrict the distribution to a low number of resilient species, the total absence of records over large areas suggests a sampling bias [80]. However, the revision of threats indicates that the coastal region has a high proportion of its surface included under the highest risk, as well as a low representation in the Protected Areas Network (Fig 4). We emphasize the need for urgent base-line information regarding the amphibians inhabiting this region, as the lack of data makes it impossible to detect and monitor potential population declines or local extinctions.

Towards a robust and objective methodology to evaluate species conservation status

The methodology implemented herein is explicit, objective, and consistent, which are the main requisites to produce a solid assessment of species conservation categories. We are confident that we have produced standardized parameters to estimate robust risk variables that integrate interacting threats [2,59]. We consider it as a key step of improving the protocol for Red List assessment in the effort to validate the taxonomic and spatial database. Ecological modeling was performed using all available data points for nominal species, and as such

included historical records, identifying and avoiding species complexes, and candidate new species based on phylogenetic evidence [36,37,40,42,81-84]. Although experts participated in the evaluation of the current status it is possible that the risk of extinction of some species is higher than assessed, due to the decline in their distribution range over time, as well as limitations on our understanding of population dynamics and ecological interactions [11,85]. Demographic information is lacking for the vast majority of Ecuadorian amphibians (Fig 2b). This constitutes a serious obstacle for obtaining a more comprehensive evaluation of their conservation status, preventing the early detection of declines. It is a particular case for Ecuador, where an important number of species are known only from a small number of specimens, and some have not been encountered for decades [e.g. 25]. This can be the result of cryptic habits that characterize some taxa (e.g. cecilians), but might as well indicate severe population declines or even extinctions (*Telmatobius, Atelopus*, or some centrolenids). This emphasizes the need for an intensive effort to gather base-line information on abundance and community composition for a diversity of amphibian populations.

Additionally, incomplete taxonomic delimitation has the potential to seriously impact amphibian conservation [71,86]. In widely distributed species complexes, which are often assessed as LC, sometimes underlie cryptic taxa [37,42,83,87], which might be facing particular conservation threats. We highlight the importance of taxonomy as a cornerstone for extinction risk assessments and conservation, especially in tropical mega-diverse regions. Assessments based on non-nominal species-level lineages or ambiguous names must be prioritized for taxonomic research [88].

Conclusions

We offer the Red List Assessment for amphibian species in Ecuador, as one of the most detailed and complete taxonomic coverage for any Ecuadorian taxonomic group to date. Our evaluation assessed that 57% of species qualified as Threatened, 12% as Near Threatened, and 4% as Data Deficient. This assessment surprisingly almost doubled the number of species considered as threatened compared to the previous evaluation in 2011 [28]. Most threatened species are widely distributed towards montane forest and paramo in the Andes, with nearly 10% of them found to occur only outside protected areas. To complement the results of this work and other future works, there is an urgent need for increasing the number of integrative taxonomic studies to describe new species and generate data on the ecology and genetics of populations and communities for those considered as taxonomic complexes. It is essential to focus research efforts on species categorized as DD, that may be in danger of extinction [30,89]. Such integration will help in better management and conservation of amphibian species in hot-spot countries, like Ecuador.

Acknowledges

We thank to Pablo Larco, Patricia Pachacama and Paola Guijarro (PARG Project), for their valuable support along the red list assessment; to Andrea Coloma, Eduardo Toral, Stephanie Arellano, Ernesto Arbeláez, Diego Inclán and Grace C. Reyes Ortega, for sharing information and technical comments along the workshops and database curation.

Financial disclosure

This work was supported by the project “Conservation of Ecuadorian Amphibians and access to genetic resources-PARG”; projects “On the quest of the golden fleece in Amazonia: The first herpetological DNA - barcoding expedition to unexplored areas on the Napo watershed, Ecuador”, funded by the Secretaría Nacional de Ciencia y Tecnología del Ecuador (Senescyt-ENSAMBLE Grant #PIC-17-BENS-001) and The World Academy of Sciences (TWAS Grant #16-095) granted to HMOA; and the project Critical Ecosystem Partnership Fund (CEPF) grant CEPF-108984 “Amphibian Conservation in the Abra de Zamora Key Biodiversity Area of Ecuador” granted to DAO, PS and DS.

References

1. Betts J, Young RP, Hilton-Taylor C, Hoffmann M, Rodríguez JP, et al. (2020) A framework for evaluating the impact of the IUCN Red List of threatened species. Conservation Biology 34: 632-643.
2. Collen B, Dulvy NK, Gaston KJ, Gärdenfors U, Keith DA, et al. (2016) Clarifying misconceptions of extinction risk assessment with the IUCN Red List. Biology Letters 12: 20150843.
3. IUCN (2017) Guidelines for Using the IUCN Red List Categories and Criteria. Version 13 Prepared by the Standards and Petitions Subcommittee of the International Union for the Conservation of Nature.
4. IUCN (2020) The UICN Red List of Threatened Species.
5. Vasconcelos TS, da Silva FR, dos Santos TG, Prado VH, Provete DB (2019) Biogeographic Patterns of South American Anurans. Switzerland: Springer International Publishing. 149 p.
6. Vitt LJ, Caldwell JP (2014) Herpetology: An introductory biology of amphibians and reptiles. London: Academic Press. 757 p.
7. Hof C, Araújo MB, Jetz W, Rahbek C (2011) Additive threats from pathogens, climate and land-use change for global amphibian diversity. Nature 480: 516-519.

8. McCain CM, Colwell RK (2011) Assessing the threat to montane biodiversity from discordant shifts in temperature and precipitation in a changing climate. Ecology Letters 14: 1236-1245.
9. Scheele BC, Pasmans F, Skerratt LF, Berger L, Martel A, et al. (2019) Amphibian fungal panzootic causes catastrophic and ongoing loss of biodiversity. Science 363: 1459-1463.
10. Ceballos G, Ehrlich P, Dirzo R (2017) Biological Annihilation via the Ongoing Sixth Mass Extinction Signaled by Vertebrate Population Losses and Declines. PNAS 114: E6089-E6096.
11. Menéndez-Guerrero PA, Davies TJ, Green DM (2020) Extinctions of Threatened Frogs may Impact Ecosystems in a Global Hotspot of Anuran Diversity. Herpetologica 76: 121-131.
12. Reider K (2018) Survival at the Summits: Amphibian Responses to Thermal Extremes, Disease, and Rapid Climate Change in the High Tropical Andes: digitalcommons.fiu.edu.
13. AmphibiaWeb (2020) AmphibiaWeb: Information on amphibian biology and conservation. 2020 ed: Berkeley, California.
14. Centro-Jambatu (2020) Anfibios de Ecuador. Fundación Otonga, Quito, Ecuador.
15. Ron SR, A. M-V, A. OD (2020) Anfibios del Ecuador. Version 2019.0. Quito, Ecuador: Museo de Zoología, Pontificia Universidad Católica del Ecuador.
16. Frost DR (2020) Amphibian Species of the World: an Online Reference. 6.1 ed. New York, USA: American Museum of Natural History.
17. Mayani-Parás F, Botello F, Castañeda S, Sánchez-Cordero V (2019) Impact of Habitat Loss and Mining on the Distribution of Endemic Species of Amphibians and Reptiles in Mexico. Diversity 11: 1-11.
18. Botello F, Sarkar S, Sánchez-Cordero V (2015) Impact of habitat loss on distributions of terrestrial vertebrates in a high-biodiversity region in Mexico. Biological Conservation 184: 59-65.
19. Cisneros-Heredia DF (2008) Habitat loss and climate change impacts on Neotropical anurans: Implications for in-situ conservation. A case study with Glassfrogs from eastern Ecuador (Amphibia: Anura: Centrolenidae). London: M.Sc. Dissertation. King's College. 73 p.
20. Lessmann J, Muñoz J, Bonaccorso E (2014) Maximizing species conservation in continental Ecuador: a case of systematic conservation planning for biodiverse regions. Ecology and Evolution 2014: 1-13.

21. Tapia-Armijos MF, Homeier J, Espinosa CI, Leuschner C, Cruz MdI (2015) Deforestation and Forest Fragmentation in South Ecuador since the 1970s – Losing a Hotspot of Biodiversity. PLOS ONE 10: e0133701.

22. Gomes VHF, Vieira ICG, Salomão RP, ter Steege H (2019) Amazonian tree species threatened by deforestation and climate change. Nature Climate Change 9: 547-553.

23. Bustamante MR, Ron SR, Coloma LA (2005) Cambios en la Diversidad en Siete Comunidades de Anuros en los Andes de Ecuador. Biotropica 37: 180–189.

24. La Marca E, Lips K, Lötters S, Puschendorf R, Ibáñez R, et al. (2006) Catastrophic Population Declines and Extinctions in Neotropical Harlequin Frogs (Bufonidae: Atelopus). Biotropica 37: 190–201.

25. Coloma LA, Duellman WE, Almendariz A, Ron S, Terán-Valdez A, et al. (2010) Five new (extinct?) species of *Atelopus* (Anura: Bufonidae) from Andean Colombia, Ecuador, and Peru. Zootaxa 2574: 1-54.

26. IUCN (2008) The IUCN Amphibians Initiative: A record of the 2001-2008 assessment efforts for the IUCN Red List.

27. Wake DB, Vredenburg VT (2008) Are we in the midst of the sixth mass extinction? A view from the world of amphibians. Proceedings of the National Academy of Sciences 105: 11466-11473.

28. Ron SR, Guayasamin JM, Menéndez-Guerrero P (2011) Biodiversity and conservation status of Ecuadorian Amphibians. In: Heatwole H, L. B-AC, Wilkinson HW, editors. Amphibian Biology Part 2. Baulkham Hills, Australia: Surrey Beatty & Soons PTY Limited. pp. 129-170.

29. González-del-Pliego P, Freckleton RP, Edwards DP, Koo MS, Scheffers BR, et al. (2019) Phylogenetic and Trait-Based Prediction of Extinction Risk for Data-Deficient Amphibians. Current Biology 29: 1557-1563.e1553.

30. Howard SD, Bickford DP (2014) Amphibians over the edge: Silent extinction risk of Data Deficient species. Diversity and Distributions 20: 837-846.

31. Grant T, Rada M, Anganoy-Criollo M, Batista A, Dias PH, et al. (2017) Phylogenetic Systematics of Dart-Poison Frogs and Their Relatives Revisited (Anura: Dendrobatoidea). South American Journal of Herpetology 12.

32. Guayasamin JM, Castroviejo-Fisher S, Trueb L, Ayarzagüena J, Rada M, et al. (2009) Phylogenetic systematics of Glassfrogs (Amphibia: Centrolenidae) and their sister taxon *Allophryne ruthveni*. Zootaxa 2100: 1-97.

33. Castroviejo-Fisher S, DE LA RIVA I, POMBAL JR JP, da Silva HR, Rojas-Runjaic FJ, et al. (2015) Phylogenetic systematics of egg-brooding frogs (Anura: Hemiphractidae) and the evolution of direct development. Zootaxa 4004: 1-75.

34. Hedges SB, Duellman WE, Heinicke MP (2008) New World direct-developing frogs (Anura: Terrarana): Molecular phylogeny, classification, biogeography, and conservation. Zootaxa 2008: 1–182.

35. Sánchez-Nivicela JC, Peloso PLV, Urgiles VL, Yánez-Muñoz MH, Sagredo Y, et al. (2020) Description and phylogenetic relationships of a new trans-Andean species of Elachistocleis Parker 1927 (Amphibia, Anura, Microhylidae). Zootaxa 4779: 18.

36. Guayasamin J, Cisneros-Heredia D, McDiarmid R, Peña P, Hutter C (2020) Glassfrogs of Ecuador: Diversity, Evolution, and Conservation. Diversity 12: 1-222.

37. Páez NB, Ron SR (2019) Systematics of *Huicundomantis*, a new subgenus of *Pristimantis* (Anura, Strabomantidae) with extraordinary cryptic diversity and eleven new species. ZooKeys 868: 1-112.

38. Urgiles VL, Székely P, Székely D, Christodoulides N, Sanchez-Nivicela JC, et al. (2019) Genetic delimitation of *Pristimantis orestes* (Lynch, 1979) and *P. saturninoi* Brito et al., 2017 and description of two new terrestrial frogs from the *Pristimantis orestes* species group (Anura, Strabomantidae). ZooKeys 864: 111-146.

39. Reyes-Puig C, Pablo Reyes-Puig J, A Velarde-Garcéz D, Nicolás D, Mancero E, et al. (2019) A new species of terrestrial frog Pristimantis (Strabomantidae) from the upper basin of the Pastaza River, Ecuador. ZooKeys 832: 113-133.

40. Carvajal-Endara S, Coloma LA, Morales-Mite MA, Guayasamin JM, Szekely P, et al. (2019) Phylogenetic systematics, ecology, and conservation of marsupial frogs (Anura: Hemiphractidae) from the Andes of southern Ecuador, with descriptions of four new biphasic species. Zootaxa 4562: 1-102.

41. Navarrete MJ, Venegas PJ, Ron SR (2016) Two new species of frogs of the genus Pristimantis from Llanganates National Park in Ecuador with comments on the regional diversity of Ecuadorian Pristimantis (Anura, Craugastoridae). Zookeys 10.3897/zookeys.593.8063: 139-162.

42. Ortega-Andrade HM, Rojas-Soto OR, Valencia JH, Espinosa de los Monteros A, Morrone JJ, et al. (2015) Insights from integrative systematics reveal cryptic diversity in *Pristimantis* Frogs (Anura: Craugastoridae) from the upper Amazon basin. PLoS One 10: e0143392.

43. Reyes-Puig C, Yánez Muñóz M, Ortega J, Ron S (2020) Relaciones filogenéticas del subgénero *Hypodictyon* (Anura: Strabomantidae: *Pristimantis*) con la descripción de tres especies nuevas de la región del Chocó. Revista Mexicana de Biodiversidad 91: 1-38.

44. Chapman AD (2005) Principles of Data Quality. Version 1.0. Copenhagen: Report for the Global Biodiversity Information Facility.
45. Barve N, Barve V, Jiménez-Valverde A, Lira-Noriega A, Maher SP, et al. (2011) The crucial role of the accessible area in ecological niche modeling and species distribution modeling. Ecological Modelling 222: 1810-1819.
46. Bedia J, Herrera S, Gutiérrez JM (2013) Dangers of using global bioclimatic datasets for ecological niche modeling. Limitations for future climate projections. Global and Planetary Change 107: 1-12.
47. Peterson AT, Soberón J (2012) Species distribution modeling and ecological niche modeling: getting the concepts right. Natureza & Conservação 10: 102-107.
48. Broennimann O, Fitzpatrick MC, Pearman PB, Petitpierre B, Pellissier L, et al. (2012) Measuring ecological niche overlap from occurrence and spatial environmental data. Global Ecology and Biogeography 21: 481-497.
49. Pearman PB, Guisan A, Broennimann O, Randin CF (2008) Niche dynamics in space and time. Trends in Ecology and Evolution 23: 149-158.
50. Fick SE, Hijmans RJ (2017) WorldClim 2: new 1-km spatial resolution climate surfaces for global land areas. International Journal of Climatology 37: 1-14.
51. Escobar LE, Lira-Noriega A, Medina-Vogel G, Townsend Peterson A (2014) Potential for spread of the white-nose fungus (Pseudogymnoascus destructans) in the Americas: use of Maxent and NicheA to assure strict model transference. Geospatial Health 9: 221-229.
52. Riahi K, Rao S, Krey V, Cho C, Chirkov V, et al. (2011) RCP 8.5—A scenario of comparatively high greenhouse gas emissions. Climatic Change 109: 33-57.
53. Elith J, Phillips SJ, Hastie T, Dudík M, Chee YE, et al. (2011) A statistical explanation of MaxEnt for ecologists. Diversity and Distributions 17: 43–57.
54. Cobos ME, Peterson AT, Barve N, Osorio-Olvera L (2019) kuenm: an R package for detailed development of ecological niche models using Maxent. PeerJ 7: e6281.
55. Qiao H, Escobar LE, Peterson AT (2017) Accessible areas in ecological niche comparisons of invasive species: Recognized but still overlooked. Scientific reports 7: 1213.
56. Morrone JJ (2014) Biogeographical regionalisation of the Neotropical region. Zootaxa 3782: 1–110.
57. IUCN (2019) Guidelines for Using the IUCN Red List Categories and Criteria. Version 14. Standards and Petitions Subcommittee of the International Union for the Conservation of Nature.
58. Tennekes M (2018) tmap: Thematic Maps in R. Journal of Statistical Software 84: 1-39.

59. Salafsky N, Salzer D, Stattersfield AJ, Hilton-Taylor C, Neugarten R, et al. (2008) A Standard Lexicon for Biodiversity Conservation: Unified Classifications of Threats and Actions. Conservation Biology 22: 897-911.

60. Pauli H, Gottfried M, Reiter K, Klettner C, Grabherr G (2007) Signals of range expansions and contractions of vascular plants in the high Alps: observations (1994?2004) at the GLORIA master site Schrankogel, Tyrol, Austria. Global Change Biology 13: 147-156.

61. Warren DL, Seifert SN (2011) Ecological niche modeling in Maxent: the importance of model complexity and the performance of model selection criteria. Ecological Applications 21: 335-342.

62. MAE (2013) Sistema de Clasificación de los Ecosistemas del Ecuador Continental. Quito, Ecuador: Ministerio del Ambiente del Ecuador, Subsecretaría de Patrimonio Natural. 232 p.

63. MAE (2020) Mapa Interactivo del Ministerio del Ambiente. Quito: Ministerio del Ambiente y Agua del Ecuador.

64. IGM (2002) Cartas topográficas del Ecuador 1: 250 000. 1.0 ed. Quito, Ecuador: Instituto Geográfico Militar.

65. Armenteras D, Espelta J, Rodríguez N, Retana J (2017) Deforestation dynamics and drivers in different forest types in Latin America: Three decades of studies (1980–2010). Global Environmental Change 46: 139-147.

66. Roy BA, Zorrilla M, Endara L, Thomas DC, Vandegrift R, et al. (2018) New Mining Concessions Could Severely Decrease Biodiversity and Ecosystem Services in Ecuador. Tropical Conservation Science 11: -20.

67. INEC (2010) Census Data Base of 2010.

68. Bässler C, Müller J, Hothorn T, Kneib T, Badeck F, et al. (2010) Estimation of the extinction risk for high-montane species as a consequence of global warming and assessment of their suitability as cross-taxon indicators. Ecological indicators 10: 341-352.

69. Menéndez-Guerrero PA, Graham CH (2013) Evaluating multiple causes of amphibian declines of Ecuador using geographical quantitative analyses. Ecography 36: 1-14.

70. Mooers AO, Faith DP, Maddison WP (2008) Converting endangered species categories to probabilities of extinction for phylogenetic conservation prioritization. PLoS One 3: 1-5.

71. Guillory W, Muell M, Summers K, Brown J (2019) Phylogenomic Reconstruction of the Neotropical Poison Frogs (Dendrobatidae) and Their Conservation. Diversity 11: 126.

72. IUCN (2012) Guidelines for application of IUCN Red List Criteria at regional and national levels. Gland, Switzerland and Cambridge: Version 4.0. IUCN Species Survival Commission. 41 p.
73. Lessmann J, Fajardo J, Bonaccorso E, Bruner A (2019) Cost-effective protection of biodiversity in the western Amazon. Biological Conservation 235: 250-259.
74. Pearman PB (1997) Correlates of amphibian diversity in an altered landscape of Amazonian Ecuador. Conservation Biology 11: 1211-1225.
75. Harcourt AH, Parks SA (2003) Threatened primates experience high human densities: adding an index of threat to the IUCN Red List criteria. Biological Conservation 109: 137-149.
76. Cruz-Cordovez C, Herrera I, Espinoza F, Rizzo K, ... (2020) New record of a feral population of Lithobates catesbeianus Shaw, 1802 in a protected area (Santay Island) in the Ecuadorian coast: reabic.net.
77. Crespo V (2015) ¡Alerta con las especies exóticas invasoras de agua dulce! Nuestra Ciencia 17: 12-16.
78. Lever C (2003) Naturalized Reptiles and Amphibians of the World. New York, USA: Oxford University Press. 344 p.
79. Kearney S, Adams V, Fuller R, Possingham H, Watson J (2020) Estimating the benefit of well-managed protected areas for threatened species conservation. Oryx 54: 276–284.
80. Monsarrat S, Boshoff AF, Kerley GIH (2019) Accessibility maps as a tool to predict sampling bias in historical biodiversity occurrence records. Ecography 42: 125-136.
81. Páez-Vacas MI, Coloma LA, Santos JC (2010) Systematics of the *Hyloxalus bocagei* complex (Anura: Dendrobatidae), description of two new cryptic species, and recognition of *H. maculosus*. Zootaxa 2711: 1-75.
82. Caminer M, Ron S (2014) Systematics of treefrogs of the *Hypsiboas calcaratus* and *Hypsiboas fasciatus* species complex (Anura, Hylidae) with the description of four new species. ZooKeys 370: 1-68.
83. Arteaga A, Pyron RA, Peñafiel N, Romero-Barreto P, Culebras J, et al. (2016) Comparative Phylogeography Reveals Cryptic Diversity and Repeated Patterns of Cladogenesis for Amphibians and Reptiles in Northwestern Ecuador. PLoS ONE 11: e0151746.
84. Chasiluisa VD, Caminer MA, Varela-Jaramillo A, Ron SR (2020) Description and phylogenetic relationships of a new species of treefrog of the Osteocephalus buckleyi species group (Anura: Hylidae). Neotropical Biodiversity 6: 21-36.

85. Zipkin EF, DiRenzo GV, Ray JM, Rossman S, Lips KR (2020) Tropical snake diversity collapses after widespread amphibian loss. Science 367: 814-816.
86. Angulo A, Icochea J (2010) Cryptic species complexes, widespread species and conservation: lessons from Amazonian frogs of the Leptodactylus marmoratus group (Anura: Leptodactylidae). Systematics and Biodiversity 8: 357-370.
87. Ortega Andrade HM, Rojas-Soto OR, Espinosa de los Monteros A, Valencia J, Read M, et al. (2017) Revalidation of *Pristimantis brevicrus* (Anura, Terrarana) with comments on cryptic diversity in a widespread Amazonian direct-developing frog. The Herpetological Journal 26: 87-103.
88. Scherz MD, Glaw F, Hutter CR, Bletz MC, Rakotoarison A, et al. (2019) Species complexes and the importance of Data Deficient classification in Red List assessments: The case of Hylobatrachus frogs. PloS one 14: 1-32.
89. Nori J, Loyola R (2015) On the Worrying Fate of Data Deficient Amphibians. PLOS ONE 10: e0125055.

Figures

Figure 1. Spatial distribution of 37,328 records from 635 species (plus *Rana catesbeiana*, as invasive species) assessed for the IUCN Red List of Ecuadorian amphibians. Details of collections, sources, and databases are provided in S2 Table.

Figure 2. IUCN Red List of amphibians from Ecuador. The number of species by (a) Categories and (b) Criteria. Categories: CR = Critically Endangered, EN = Endangered, VU = Vulnerable, NT = Near Threatened, LC = Least Concern, DD = Data Deficient, NE = Not Evaluated - corresponds to *Rana catesbeiana*, an invasive species in Ecuador. *Atelopus ignescens* (Critically Endangered) was believed to be extinct until its rediscovery in 2016. Illustration by PARG.

Figure 3. A taxonomic perspective of the Red List status of amphibians in Ecuador. The species composition (% of threatened species) of each family in Anura (dark blue), Caudata (bright blue) and Gymnophiona (purple) is characterized by ribbons connected to the current Red List status for each species. The numerical values below each country name depict the relative percentage with the associated Red List category: CR = Critically Endangered, EN = Endangered, VU = Vulnerable, NT = Near Threatened, LC = Least Concern, and DD = Data Deficient. Two endemic and threatened frogs are illustrated for *Atelopus coynei* (Critically endangered) distributed in northern Andes of Ecuador, whereas *Excidobates condor* (Endangered) is distributed in the Cordillera del Condor, southeastern Ecuador. Both species are threatened by habitat loss, mining and climate change. Illustrations by PARG.

Figure 4. High resolution (30 m x 30 m) Environmental Risk Surface (ERS) model for Ecuadorian amphibians. Values of the ERS range from 0 (Green, low) to 1 (Red, high) to represent threat intensity. Shaded areas correspond to the National System of Protected areas shown in Fig 1.

Figure 5. Major threats associated with amphibian taxa (% of locality records in database) by conservation categories in Ecuador. Environmental contractions on climate change scenarios for RPC4.5 and RPC 8.5 are shown for those species with more than 50% of shift.

Figure 6. Cumulative species richness for threatened taxa (*n* = 265 models) by Red List category. Maps with cumulative species (Num sp) models per category and family are shown in S3-5 Figures.

Figure 7. Occurrence data of threatened Ecuadorian amphibians by (a) taxonomic families, (b) endemic taxa, and (c) Red List categories in an altitudinal gradient. Risk categories: CR = Critically Endangered, EN = Endangered, VU = Vulnerable, NT = Near Threatened, DD = Data Deficient. Least Concern taxa have been removed.

Figure 8. Occurrence data of NT (green), DD (grey), and NE (blue) Ecuadorian amphibian species, by Red List category and family. Only families with species in these categories are shown. Boana picturata (NT) is an inhabitant of the Chocoan region in northwestern Ecuador, threatened by habitat loss and fragmentation.

Figure 9. Frequency of locality records of amphibians in each risk category by Natural Regions in Ecuador. Categories: CR = Critically Endangered, EN = Endangered, VU = Vulnerable, NT = Near Threatened, DD = Data Deficient. Least Concern taxa have been removed from this figure.

Figure 10. The IUCN Red List of amphibians from Ecuador representation in the National System of Protected Areas. Categories: CR = Critically Endangered, EN = Endangered, VU = Vulnerable, NT = Near Threatened, LC = Least Concern, DD = Data Deficient. SNAP – Governmental National System of Protected Areas, from the Spanish acronym.

Tables

Table 1. Major threats with their subcategories, influence distance, decay function, and Analytic Hierarchy Process (AHP) intensity value estimated for modeling threats to Ecuadorian amphibians.

Major threats and categories	Influence Distance (m)	Decay function	AHP Intensity
**Agriculture and aquaculture**			
Crops			
Permanent crops	1875	Logistic	0.015
Annual crops	1250		0.023
Semi-permanent crops	375		0.016
Grassland	375		0.036
Agricultural mosaic	375		0.003
Forest plantations	250		0.007
Other agricultural lands	125		0.005
Aquaculture			
Shrimp farm area	1250	MSSmall	0.051
**Biological resource use**			
Deforestation	125	Logistic	0.34
**Emerging diseases and Invasive species**			
Fungus *Chytridium*	1250	Constant	0.035
Rana catesbeiana (Bullfrog)	1250	Constant	0.012
**Energy production and mining**			
Operations	1250	MSSmall	0.026
Explorations	1000		0.005
Mining and quarrying			
Concessions	625		0.002
Construction Materials/Free use/Artisanal mining	250		0.007
Oil drilling			
Active oil fields	1250	Logistic	0.015
Oil wells	625		0.021
Dormant oil fields	250		0.004
Oil blocks	250		0.003
Hydroelectric power plants			
operative	1250		0.009
Building	625	MSSmall	0.012
In project	250		0.001
**Natural system modifications**			
Megaprojects area of influence	1250	MSSmall	0.033
**Population density**			
Population density	Continuous raster	Continuous	0.22
**Transportation**			
1st order	1250	Lineal	0.025
2nd order	1000		0.016
3rd order	625		0.011
Roads			
Trails	250		0.008
Airports			
Airports	1250	Logistic	0.006
Airport runways	625		0.003
Oil pipeline/Polyduct	625	Logistic	0.002
Pipelines			
Gas pipeline	250		0.002
**Stochastic events**			
Flood-prone areas	625	MSSmall	0.005
Volcanism area of influence	12500	MSSmall	0.016

Table 2. Species and records by conservation categories in a database for Red List Assessment. CR = Critically Endangered, EN = Endangered, VU = Vulnerable, NT = Near Threatened, LC = Least Concern, DD = Data Deficient, NE = No Evaluated, corresponds to *Rana catesbeiana*, an invasive species in Ecuador.

Collections Databases	CR	EN	VU	NT	LC	DD	NE*	Species (%)	Records (%)
Global Biodiversity Information Facility	70 (702)	107 (715)	98 (1320)	64 (1201)	162 (11313)	19 (122)	1 (3)	521 (82%)	15376 (41%)
BIOWEB-PUCE	47 (239)	114 (1216)	101 (1889)	68 (2065)	168 (10161)	10 (211)	1 (2)	509 (80%)	15783 (42%)
Instituto Nacional de Biodiversidad	18 (29)	81 (238)	91 (314)	59 (321)	152 (1883)	6 (31)		407 (64%)	2816 (8%)
Museo de Zoología Universidad del Azuay	4 (6)	10 (56)	28 (169)	13 (43)	84 (262)		1 (5)	140 (22%)	541 (1%)
Museo de Zoología, Universidad Técnica Particular de Loja	8 (16)	14 (71)	31 (496)	21 (320)	57 (956)			131 (21%)	1859 (5%)
Red List Assessment Workshop	8 (25)	22 (58)	13 (40)	14 (29)	40 (145)	4 (9)	1 (1)	102 (16%)	307 (1%)
Centro Jambatu	8 (16)	14 (20)	17 (21)	10 (51)	32 (65)	()		81 (13%)	173 (0%)
Escuela Politécnica Nacional	5 (17)	8 (12)	11 (29)	10 (19)	19 (65)	1 (1)		54 (8%)	143 (0%)
Fundación Herpetológica Gustavo Orcés	1 (1)	3 (3)	1 (1)	7 (16)	16 (64)	1 (1)		29 (5%)	86 (0%)
Batrachia		3 (30)	4 (13)	1 (1)	2 (21)			10 (2%)	65 (0%)
Museo de Zoología Universidad Tecnológica Indoamérica			2 (2)	2 (2)	4 (4)	1 (1)		9 (1%)	9 (0.02%)
Literature review	2 (2)							2 (0.3%)	2 (0.01%)
Proyecto PARG	2 (168)							2 (0.3%)	168 (0.5%)
**Total Species (records)**	**85 (636)**	**147 (2419)**	**131 (4294)**	**78 (4068)**	**26 (24939)**	**168 (376)**	**1 (11)**		**37328**

Table 3. Species (percentage) and categories of risk, assessed by a family in Ecuadorian amphibians. CR = Critically Endangered, EN = Endangered, VU = Vulnerable, NT = Near Threatened, LC = Least Concern, DD = Data Deficient. Pale red-shaded numbers are highlighted for families with the highest number of species in each threatened category. Threatened representativeness (TR): (number of threatened taxa / total number of taxa per family)*100.

Class/Families	CR	EN	VU	NT	LC	DD	Threatened Taxa	Total Taxa	TR (%)
**Anura**	**81 (13.5%)**	**136 (22.6%)**	**124 (20.6%)**	**78 (13%)**	**162 (27%)**	**20 (3.3%)**	**341 (56.7%)**	**601 (100%)**	**56.7%**
Aromobatidae			2 (0.3%)		4 (0.7%)	1 (0.2%)	2 (0.3%)	7 (1.2%)	28.6%
Bufonidae	29 (4.8%)	7 (1.2%)	6 (1%)		12 (2%)	2 (0.3%)	42 (7%)	56 (9.3%)	75%
Centrolenidae	8 (1.3%)	22 (3.7%)	8 (1.3%)	7 (1.2%)	11 (1.8%)	4 (0.7%)	38 (6.3%)	60 (10%)	63.3%
Ceratophryidae			1 (0.2%)		1 (0.2%)	1 (0.2%)	1 (0.2%)	3 (0.5%)	33.3%
Craugastoridae					1 (0.2%)			1 (0.2%)	0%
Dendrobatidae	10 (1.7%)	12 (2%)	9 (1.5%)	9 (1.5%)	7 (1.2%)		31 (5.2%)	47 (7.8%)	66%
Eleutherodactylidae		1 (0.2%)			1 (0.2%)		1 (0.2%)	2 (0.3%)	50%
Hemiphractidae	7 (1.2%)	8 (1.3%)	1 (0.2%)	7 (1.2%)	2 (0.3%)		16 (2.7%)	25 (4.2%)	64%
Hylidae	5 (0.8%)	14 (2.3%)	6 (1%)	18 (3%)	55 (9.2%)	2 (0.3%)	25 (4.2%)	100 (16.6%)	25%
Leptodactylidae	1 (0.2%)	2 (0.3%)	2 (0.3%)	2 (0.3%)	18 (3%)		5 (0.8%)	25 (4.2%)	20%
Microhylidae		3 (0.5%)	2 (0.3%)	1 (0.2%)	5 (0.8%)	1 (0.2%)	5 (0.8%)	12 (2%)	41.7%
Pipidae					1 (0.2%)			1 (0.2%)	0%
Ranidae				1 (0.2%)	2 (0.3%)			3 (0.5%)	0%
Strabomantidae	18 (3%)	67 (11.1%)	87 (14.5%)	33 (5.5%)	42 (7%)	9 (1.5%)	172 (28.6%)	256 (42.6%)	67.2%
Telmatobiidae	3 (0.5%)						3 (0.5%)	3 (0.5%)	100%
**Caudata**	**3 (27.3%)**	**5 (45.5%)**			**2 (18.2%)**	**1 (9.1%)**	**8 (72.7%)**	**11 (100%)**	**72.7%**
Plethodontidae	3 (27.3%)	5 (45.5%)			2 (18.2%)	1 (9.1%)	8 (72.7%)	11 (100%)	72.7%
**Gymnophiona**	**1 (4.3%)**	**6 (26.1%)**	**7 (30.4%)**		**4 (17.4%)**	**5 (21.7%)**	**14 (60.9%)**	**23 (100%)**	**60.9%**
Caeciliidae		5 (21.7%)	4 (17.4%)		3 (13%)	4 (17.4%)	9 (39.1%)	16 (69.6%)	56.3%
Rhinatrematidae	1 (4.3%)	1 (4.3%)	1 (4.3%)				3 (13%)	3 (13%)	100%
Siphonopidae			1 (4.3%)		1 (4.3%)		1 (4.3%)	2 (8.7%)	50%
Typhlonectidae			1 (4.3%)			1	1 (4.3%)	2 (8.7%)	50%
**Total general**	**85 (13.4%)**	**147 (23.1%)**	**131 (20.6%)**	**78 (12.3%)**	**168 (26.5%)**	**26 (4.1%)**	**363 (57.2%)**	**635 (100%)**	**57.2%**

Supplementary Tables

S1 Table. Institution Code, Institution name, database source, categories, number of records, and number of species assessed in the Red List for Ecuadorian amphibians.

S2 Table. Multi-Criteria Decision Making (MCDM) through the Analytic Hierarchy Process (AHP) for construct the threat model (Fig 4).

S3 Table. Species list of Ecuadorian amphibians, endemism, conservation areas, major threats, extinction risk criteria, subcriteria, and metrics used for the Red List Assessment.

S4 Table. The Red List for Ecuadorian amphibians, with details of criteria and subcriteria used for the evaluation of national categories. Categories: CR= Critically Endangered, EN=Endangered, VU= Vulnerable, NT= Near Threatened, LC= Least Concern, DD= Data Deficient, NE= Not Evaluated, correspond to *Rana catesbeiana*, an invasive species in Ecuador.

S5 Table. The number of taxa assessed by genera in the current evaluation, categories, and threatened representativeness in the group (%).

S6 Table. Species (percentage) and categories of threat assessed by type of protected area in Ecuador. CR= Critically Endangered, EN=Endangered, VU= Vulnerable, NT= Near Threatened, LC= Least Concern, DD= Data Deficient. SNAP= National System of Protected Areas, from the Spanish acronym.

S7 Table. Species (percentage) and categories of the conservation status of amphibians by major threats in Ecuador. CR= Critically Endangered, EN=Endangered, VU= Vulnerable, NT= Near Threatened, LC= Least Concern, DD= Data Deficient.

S8 Table. Species and categories of threat assessed by Natural Regions and Protected Area in Ecuadorian Amphibians. CR= Critically Endangered, EN=Endangered, VU= Vulnerable, NT= Near Threatened, LC= Least Concern, DD= Data Deficient.

S9 Table. Species (percentage) and categories of threat assessed by natural regions and provinces in Ecuadorian Amphibians. CR= Critically Endangered, EN=Endangered, VU= Vulnerable, NT= Near Threatened, LC= Least Concern, DD= Data Deficient.

Supplementary figures

S1 Fig. Automated procedure was designed using the *ModelBuilder* tool in ArcMap v.10 to perform the iterative threat model and its analysis.

S2 Fig The threat model for Ecuadorian amphibians, raster image (.tiff).

https://drive.google.com/file/d/1wkdx8DgDwKhVEyElDhc23wmEiknFw4DE/view?usp=sharing

S3 Fig. Cumulative richness models of taxa qualified as Critically endangered by family.

S4 Fig Cumulative richness models of taxa qualified as Endangered by family.

S5 Fig Cumulative richness models of taxa qualified as Vulnerable by family.

Figure 1

Click here to access/download;Figure;Fig1.eps

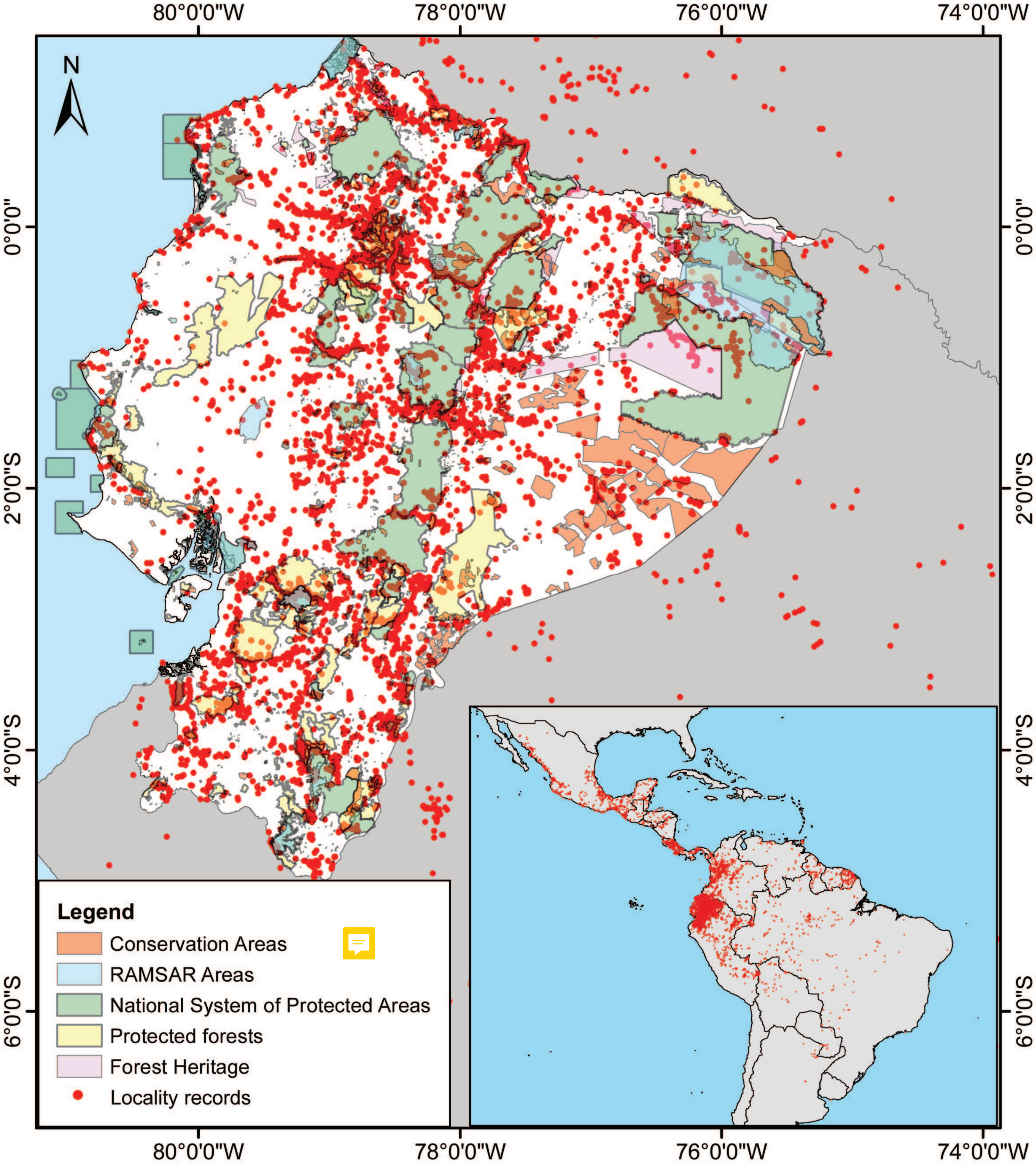

Figure 2 Click here to access/download;Figure;Fig2.tiff

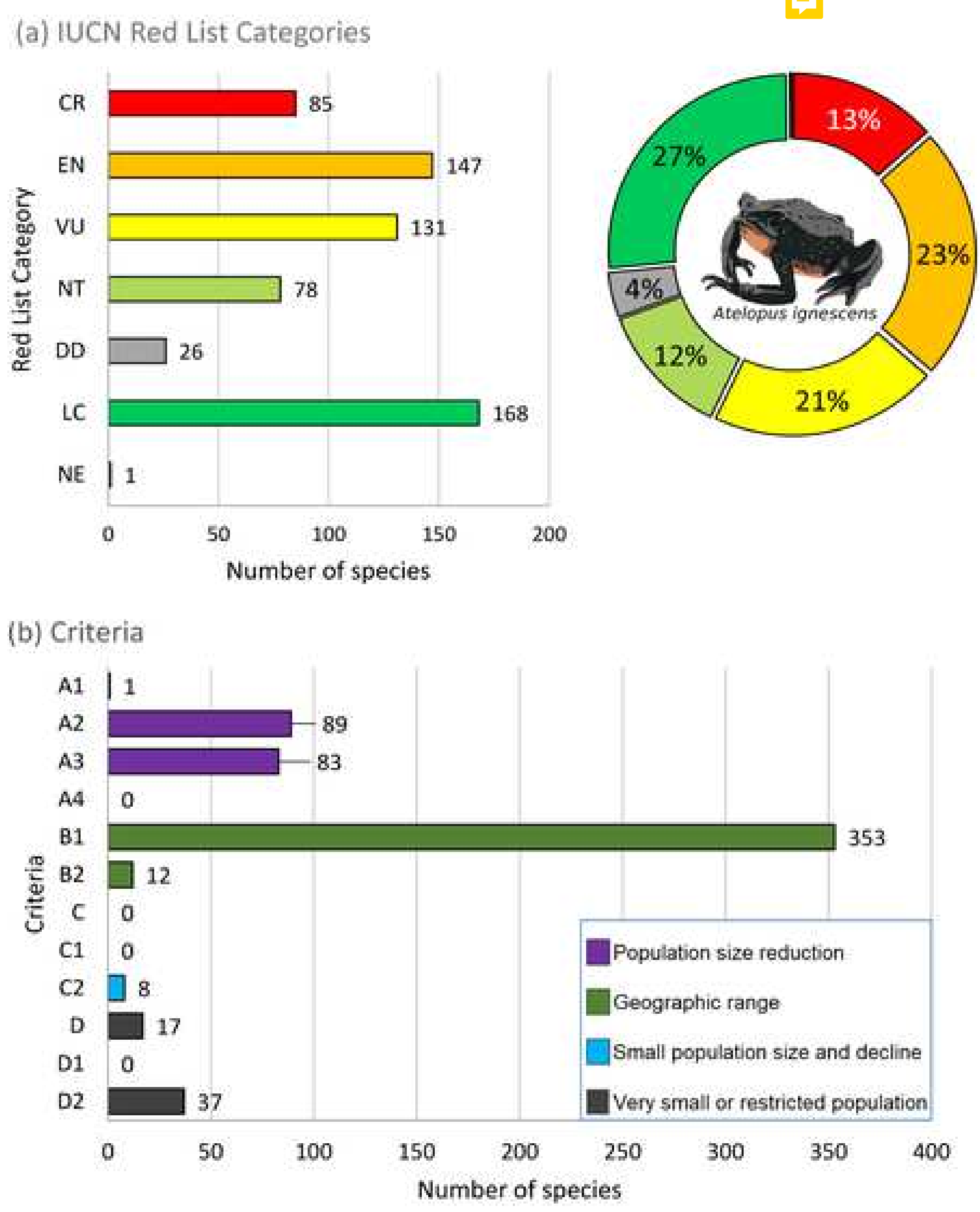

Figure 3

Click here to access/download;Figure;Fig3.eps

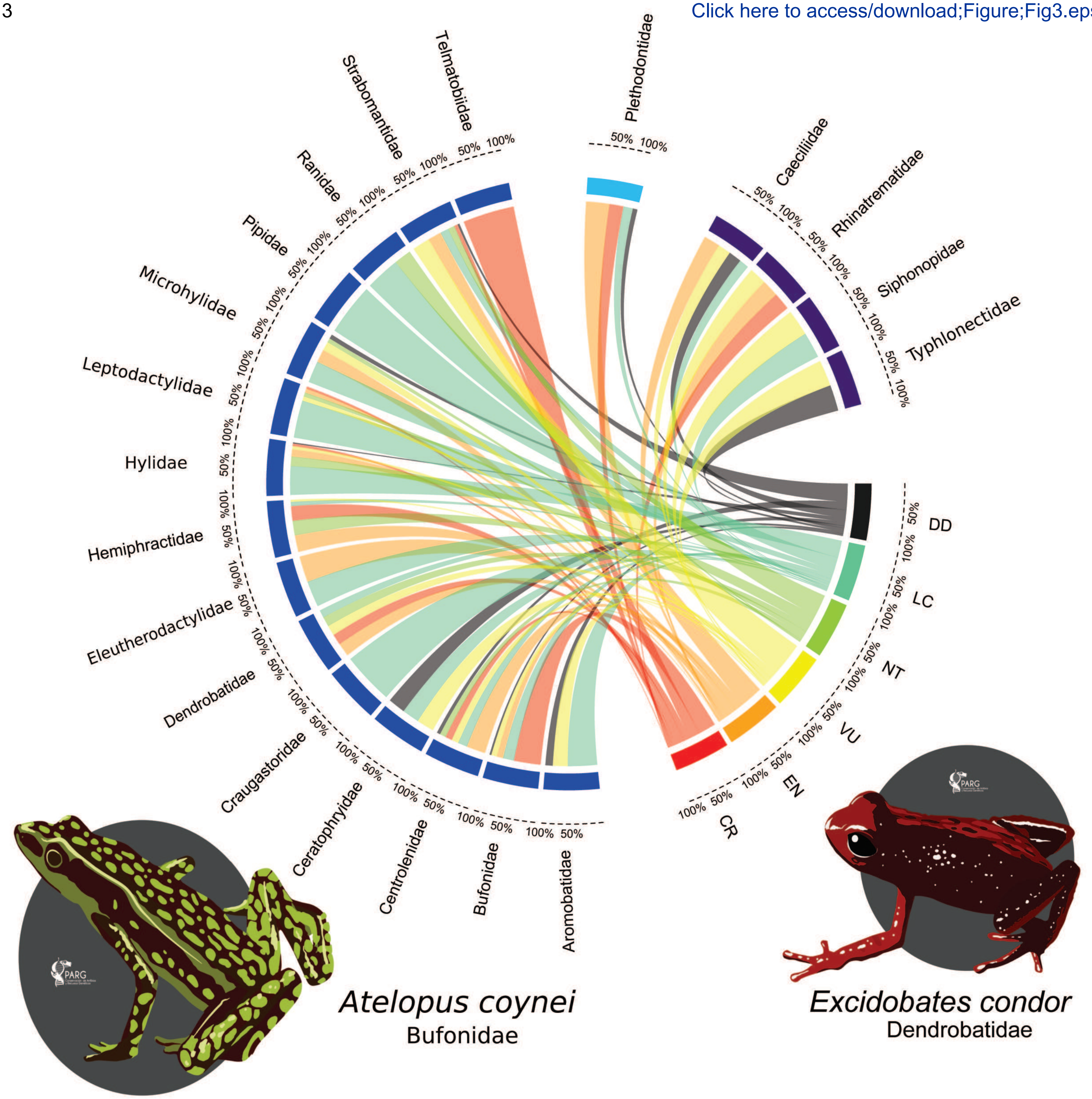

Figure 4

Click here to access/download;Figure;Fig4.eps

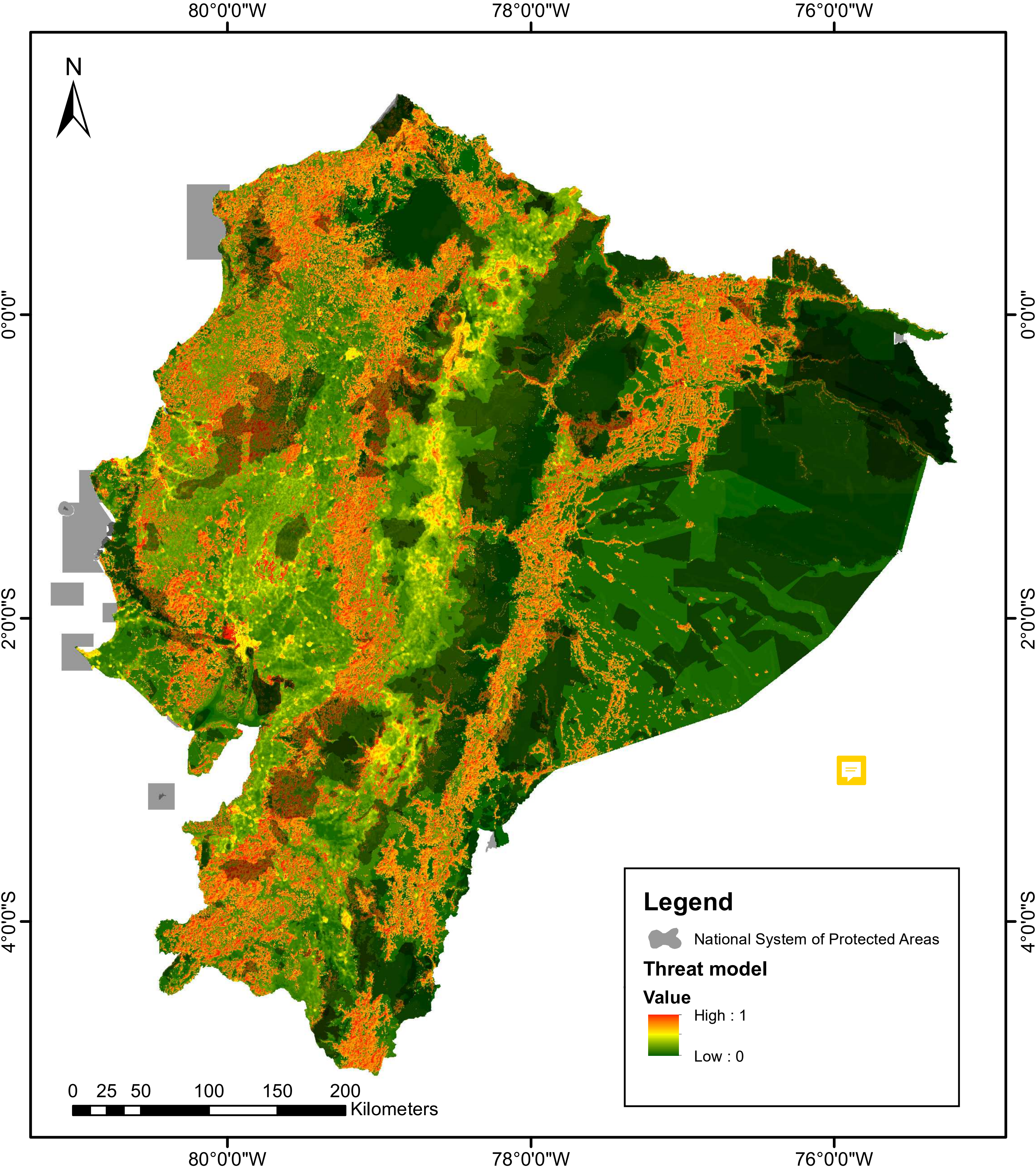

Figure 5

Click here to access/download;Figure;Fig5.eps

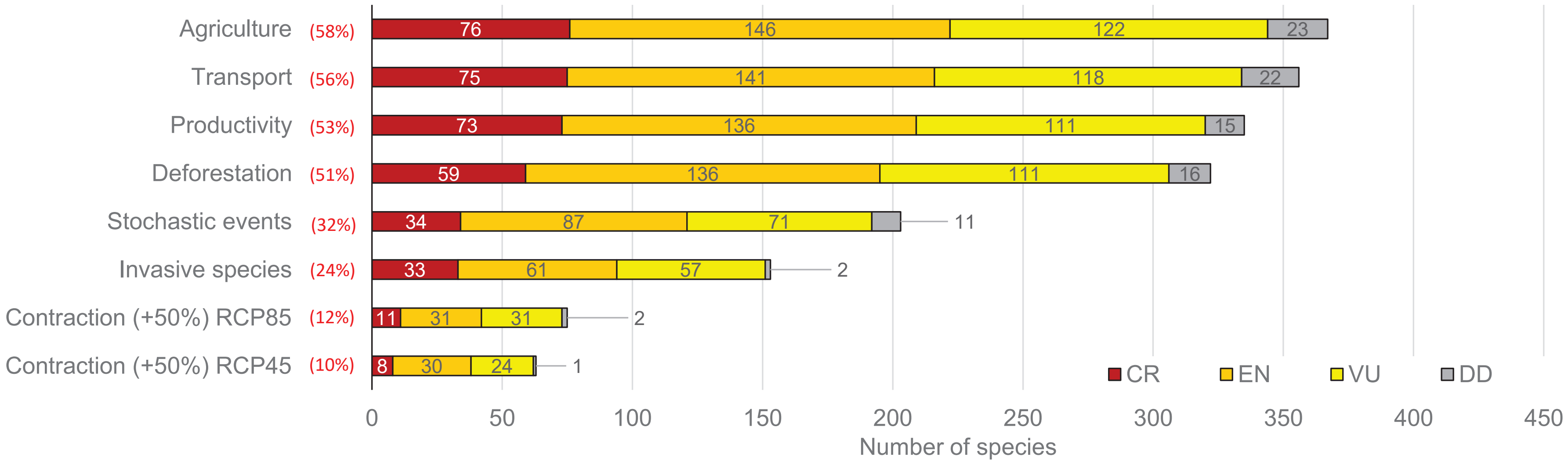

Figure 6

Click here to access/download;Figure;Fig6.eps

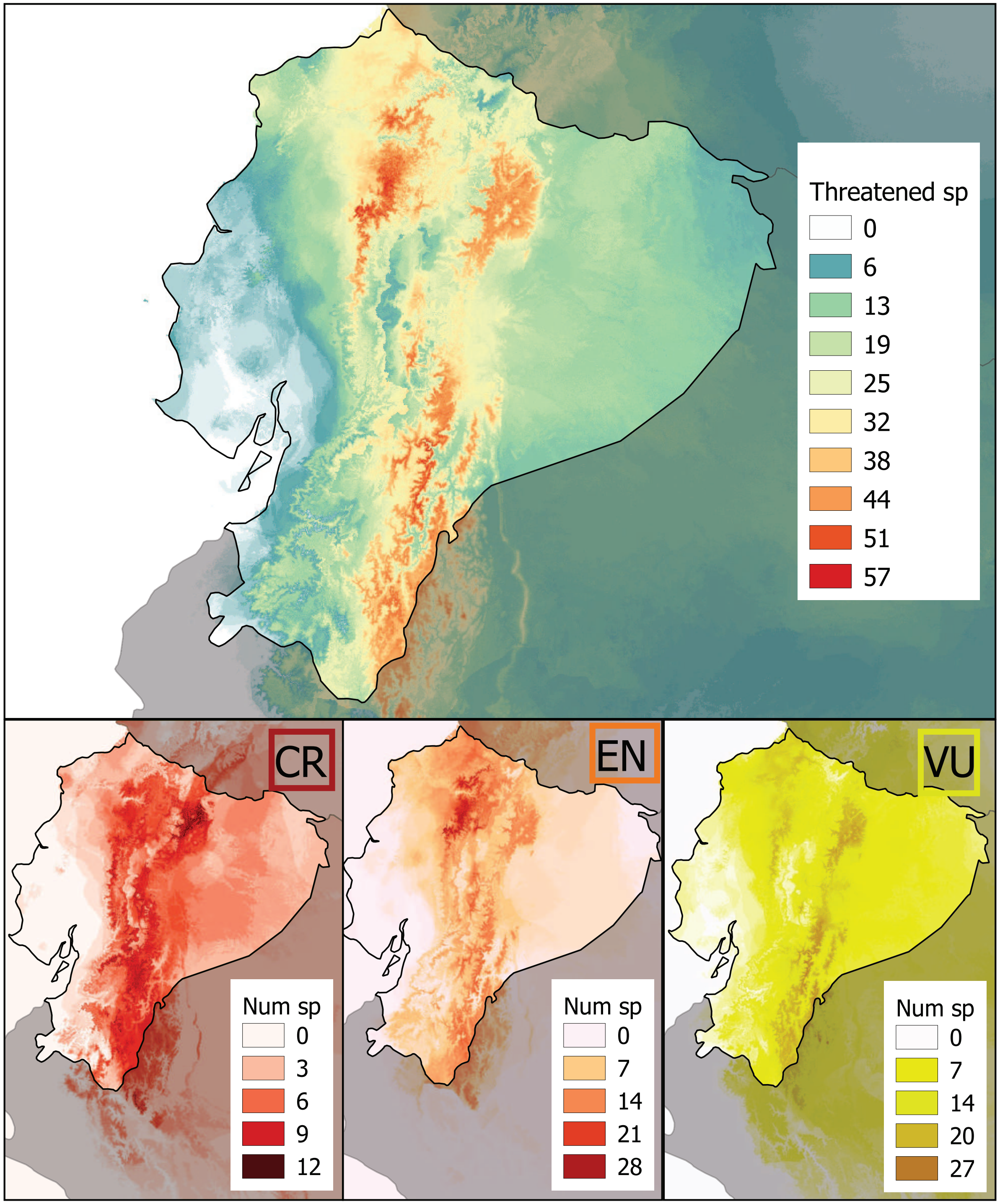

Figure 7 Click here to access/download;Figure;Fig7.eps

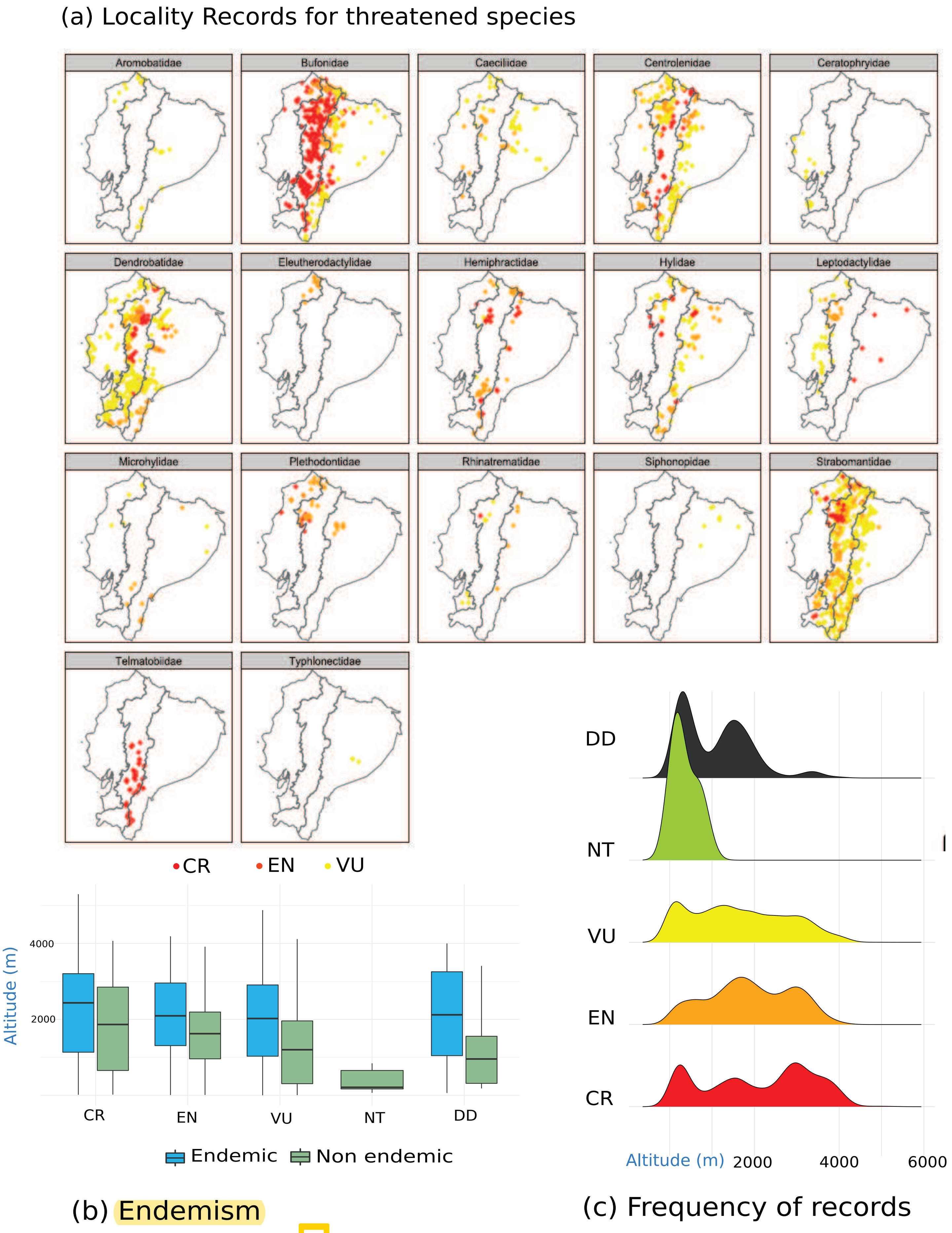

Figure 8

Click here to access/download;Figure;Fig8.eps

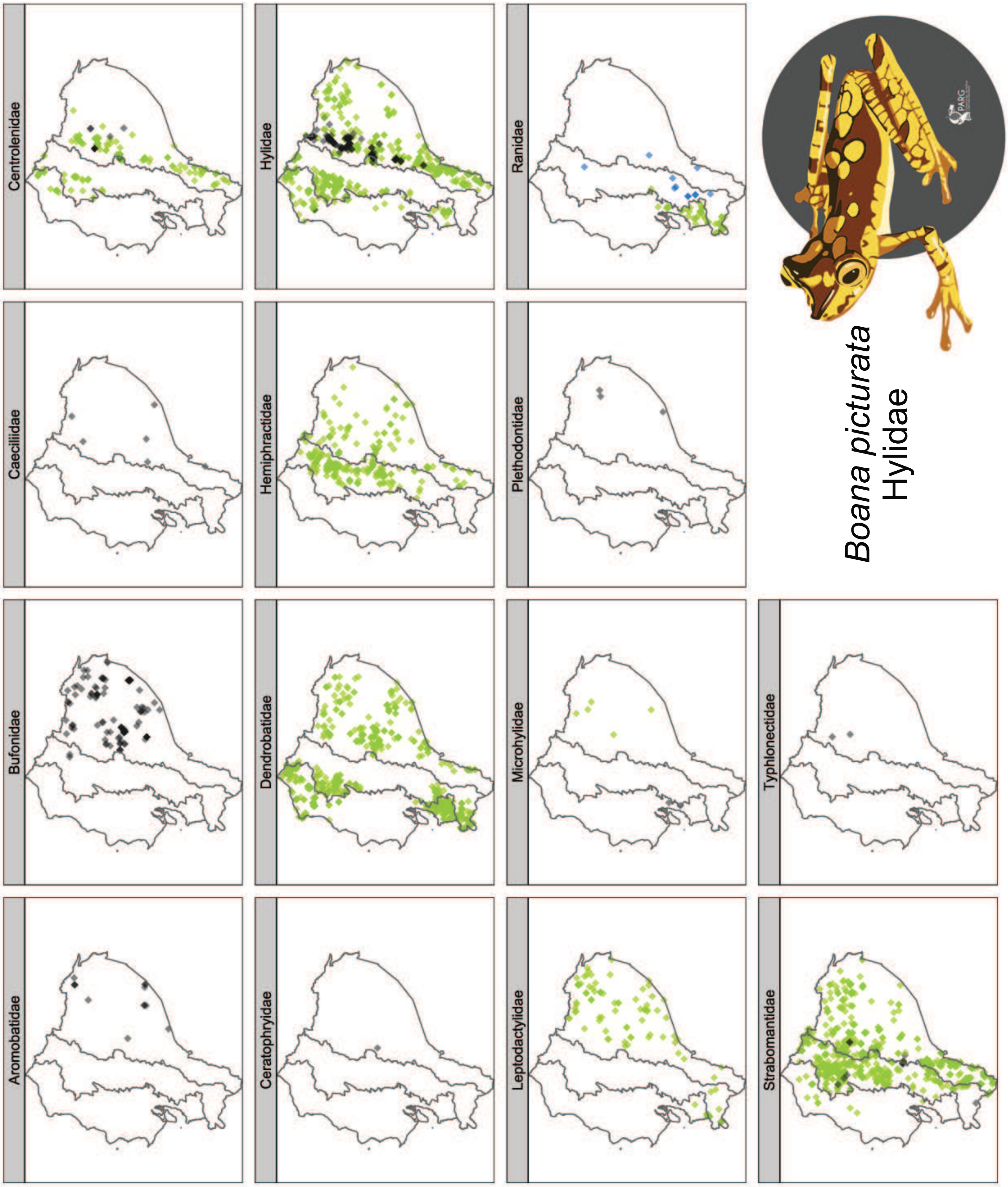

Figure 9

Click here to access/download;Figure;Fig9.eps

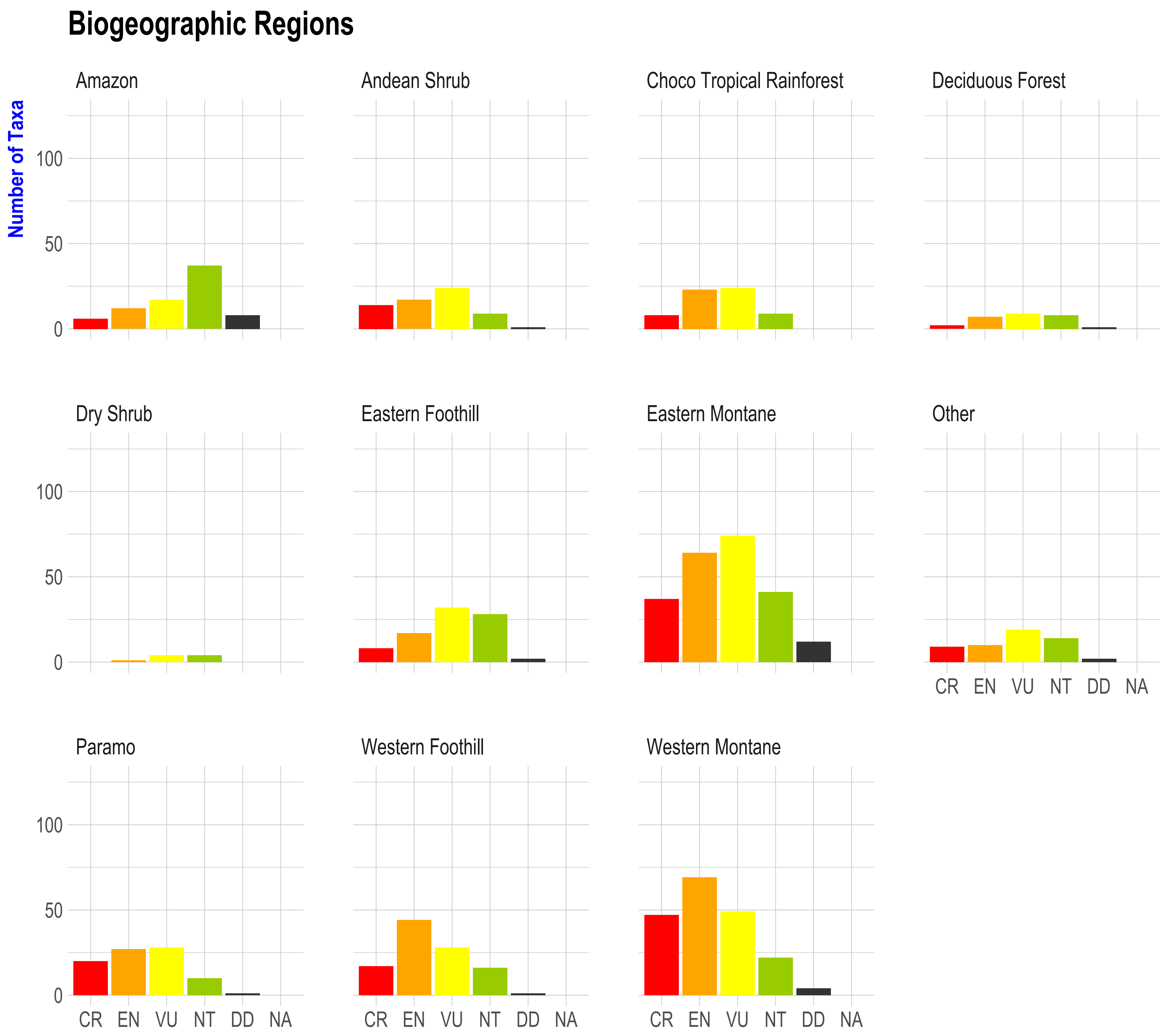

Figure 10

Click here to access/download;Figure;Fig10.eps

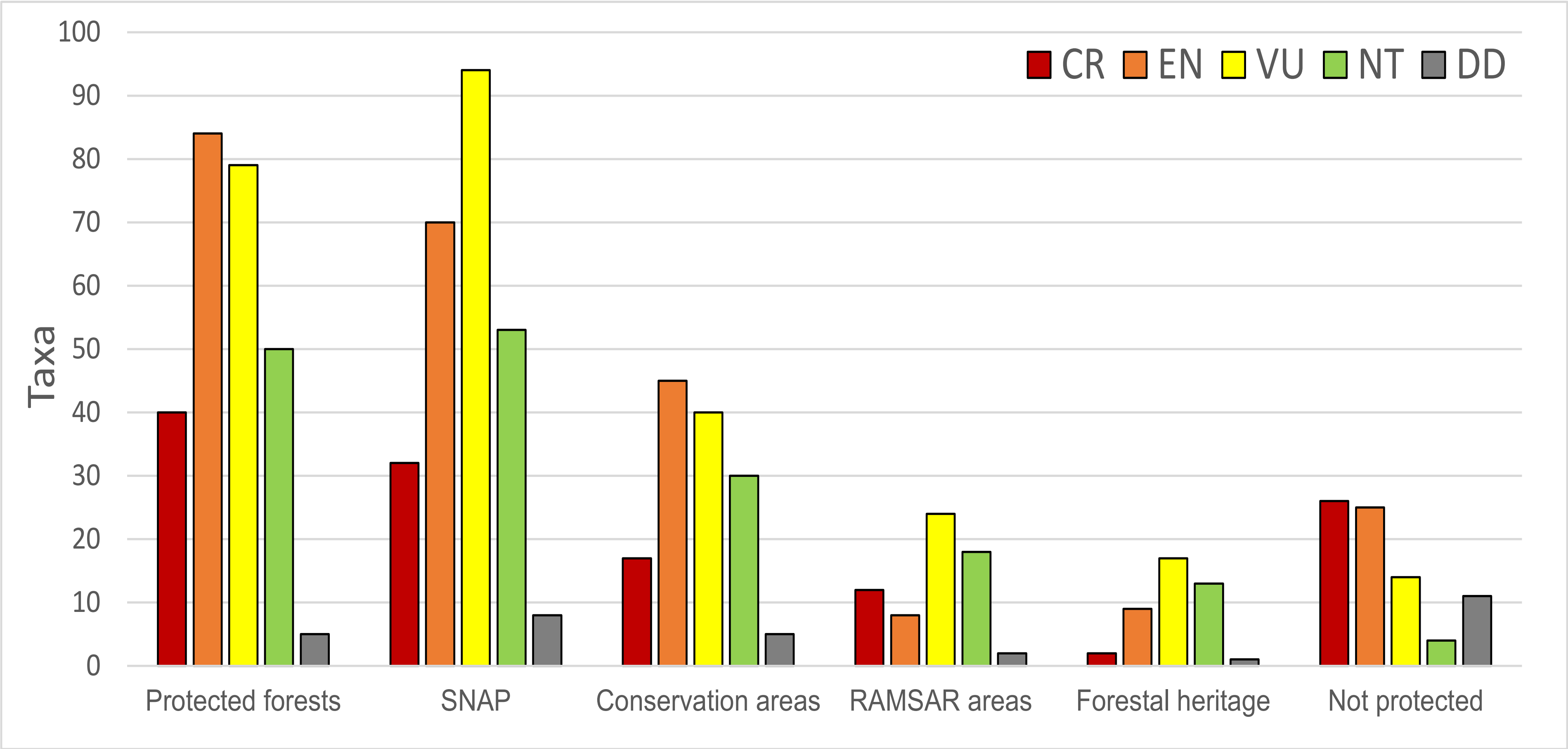

S1 Figure

Click here to access/download
Supporting Information
S1 Fig.docx

S2 Figure

Click here to access/download
Supporting Information
S2 Fig.docx

S3 Figure

Click here to access/download

Supporting Information

S3 Fig.docx

S4 Figure

Click here to access/download
Supporting Information
S4 Fig.docx

S5 Figure

Click here to access/download
Supporting Information
S5 Fig.docx

S1 Table

Click here to access/download
Supporting Information
S1 Table.xlsx

S2 Table

Click here to access/download

Supporting Information

S2 Table.xlsx

S3 Table

Click here to access/download
Supporting Information
S3 Table.xlsx

S4 Table

Click here to access/download
Supporting Information
S4 Table.xlsx

S5 Table

Click here to access/download
Supporting Information
S5 Table.xlsx

S6 Table

Click here to access/download
Supporting Information
S6 Table.xlsx

S7 Table

Click here to access/download
Supporting Information
S7 Table.xlsx

S8 Table

Click here to access/download
Supporting Information
S8 Table.xlsx

S9 Table

Click here to access/download
Supporting Information
S9 Table.xlsx
