# Supplementary material for: Red List assessment of amphibian species of Ecuador: A multidimensional approach for their conservation
Source: PLoS One. 2021 May 6;16(5):e0251027. doi: 10.1371/journal.pone.0251027 (PMC8101765; doi:10.1371/journal.pone.0251027)
Supplement: Attachment — Submitted filename: review plos paper.docx [file pone.0251027.s016.docx]

Manuscript: Red List assessment for amphibian species of Ecuador: a multidimensional approach for their conservation

Reviewer: Luis Fernando Marin da Fonte (IUCN Amphibian Specialist Group for Brazil, Amphibian Survival Alliance)

In this paper, the authors provide a very detailed and complete assessment of the conservation status of amphibians in Ecuador besides providing the most up to date list of species for the country. It is a very impressive and important work with great potential to benefit the conservation of this group of animals. Besides having a local importance, with the potential to inform public policies, it can also serve as a role model to other countries globally. I congratulate the authors for their work and recommend its publication with minor changes. That said, I have some suggestions to improve the overall quality of the manuscript and think that, before publication, some important issues should be addressed, as following:

1) with regard to the writing, I acknowledge that the authors are not native English speakers and admire their willingness to publish in a foreign language. Since I am also not a native English speaker, I cannot comment much on the language (though I have made some minor suggestions). The introduction reads very well, but the discussion reads a bit difficult sometimes, with too long and often confusing sentences. I suggest using shorter sentences to improve clarity.

2) my main criticism to this paper (though relatively easy to fix) is that it is not clear in the manuscript if this is a global or a regional/national assessment. Moreover, it is a bit confusing what are the ties of this assessment with the IUCN.

Global assessments (published on the IUCN Red List website) take into account the complete geographic range of taxa, while regional assessments take into account occurrence inside a specific region (usually inside country boundaries). Of course, for species endemic to a given country, global assessments will match national assessments, but this is not the case for species shared by different countries. The IUCN provides specific guidelines for regional assessments, but it is not clear if the authors followed them.

All things considered, one would imagine this is indeed a national assessment. However, things get a bit confusing when authors say that they have used the ‘complete distributional range’ of species (line 181). This would not be a problem per se, but it is not clear in the manuscript if the records from other countries were validated by experts - in the same way that records from inside Ecuador were validated by Ecuadorian experts in the workshops. Moreover, it is not clear if the search for records outside Ecuador was exhaustive or not (if I got it right, they were gathered only from online databases?). From what I have understood, records from outside Ecuador were not collected and analysed with the same level of accuracy than the records from inside Ecuador. If this is the case, you would have two kind of data: high quality data from inside Ecuador, and possibly incomplete/unreviewed/not validated data from outside Ecuador. If this is a regional/national assessment, this should not be a problem, since the most relevant data for the evaluations are the ones from inside Ecuador. But if this was intended to be a global assessment, then we would have a problem. In this sense, I think it is fundamental to make it clear, both in the title and in the body of the text, that (or if) this is a national assessment. Moreover, it is important to make clear if data from outside Ecuador was validated by experts and if the search for records was exhaustive (in other words, is this data comparable with the data from inside Ecuador?). Finally, it is important to show how exactly the data from outside Ecuador was used, since this is fundamental to determine if this is a global or a regional/national assessment.

Still in this topic, it is not clear if the authors followed the IUCN guidelines or only the IUCN criteria and categories. This might seem perfumery, but it is not. The IUCN guidelines inform how the criteria and categories should be used, and this is even more relevant in regional assessments. The criteria, in their turn, inform if and how the taxa fall under the different threat categories. In this sense, since the criteria and categories are the same for global and regional assessments, but the guidelines are not, it is not informative enough to only say that the assessment followed “IUCN criteria” (lines 68, 298), “IUCN standards” (lines 298, 459) or “IUCN protocols” (line 298), whatever ‘standard’ and ‘protocol’ mean in this context. It is important to know if the assessment followed the IUCN guidelines and, even more important, if it followed the guidelines for regional assessments.

Finally, it is important to make clear if this is an official IUCN assessment (i.e. if members of the IUCN Amphibian Red List Authority were present in the workshops and if the assessment was validated by external members of the IUCN Red List Authority). I specifically mention this because in line 330 the authors say “… the IUCN Red List assessment resulted in…”. If this is not an official IUCN assessment, you cannot say that. In this case, I suggest changing for “... the assessment following the IUCN Red List guidelines and criteria resulted...”. Same thing in line 151.

I also suggest reviewing the references to all IUCN documents. They are generally incomplete in the list of references and/or cited without criteria in the body of the text. For instance, the authors do cite the IUCN guidelines for regional assessments (ref. 72), though in a different context that I would expect (line 488), but do not cite it when explaining how the assessments were conducted (line 298). In line 298, the authors wrongly cite the IUCN Red List itself (ref. 4, incomplete), which informs nothing about the process, but do not cite the IUCN guidelines (ref. 3, 57 and 72). For consistency, I would also recommend avoiding using different versions of the guidelines (ref. 3, 57 and 72) throughout the manuscript – unless they deal with different and specific topics.

3) I suggest using the term ‘endemic’ in a more consistent way throughout the entire manuscript. In many cases, it is not clear if authors mean ‘endemic to Ecuador’ (what it is especially relevant if this is a national assessment) or ‘endemic to a specific region inside Ecuador’.

4) I suggest improving the discussion on the differences between the present and previous assessments. The authors say: “The differences are probably due to broader knowledge, including taxonomic revisions and species descriptions, but also to the different assessment procedures”. This is too superficial and must be better explained.

First thing to note: are both assessments really comparable? Are they both regional/national or global assessments? Did both assessments use data only from inside Ecuador or also from other countries? Did both assessments use the same guidelines? What are the “different assessment procedures” referred by the authors? Which assessment did not follow the IUCN guidelines? This must be specified. Once this is clarified, one can try to understand why there were so many differences.

DD species, for instance. What has changed since the last assessment that allowed the authors to assess 127 species that were previously considered DD? Did the knowledge about these species increased so much over the past 10 years? Or did the definition of 'sufficient' and 'insufficient' 'information to be evaluated' that in fact change? This is especially relevant to note if we take into account that the authors of both assessments are not the same.

Further information and discussion are also needed for taxa that are now listed in higher and lower categories. Again, what explains these differences? Different methods and approaches? This should not be the case, at least in theory, if both assessments followed the IUCN guidelines. Even though there is room for subjectivity and personal judgements in the application of IUCN criteria, this alone should not be enough to explain such big differences between both assessments (139 taxa). Possible explanations for it could be: a) one of the assessments did not (correctly) follow the IUCN guidelines (and that is why it is so important to stress out if and which guidelines were followed in this work); b) scientific knowledge has greatly improved over the past 10 years, allowing for much more refined and detailed assessments; c) over the past 10 years, the overall environmental conditions have improved in some regions (allowing populations/species previously under threat to recover) and decreased in others (threatening populations/species that were in a better situation when the previous assessment was conducted). If this is really the case, it is especially relevant and would be, in my view, the main finding of this study.

Anyway, I guess the most plausible explanation for the differences between both assessments is the combination of all these factors (and maybe others). But authors must discuss this better in the paper and try to provide more detailed explanations. Providing specific examples to illustrate these cases would be especially important.

5) In the section ‘**Towards a robust and objective methodology to evaluate species conservation status**’, authors suggest their methodology has the potential to improve red list assessments. What are the specific improvements/developments of your methodology with relation to others? How can other evaluators replicate your methodology? In my view, the methods are not described in the manuscript in such a detailed way that it would allow easy and straightforward adoption in other assessments. I acknowledge that the authors have used a robust methodology with the potential to improve future evaluations. But I am not sure if this manuscript, in the way that it is presented, provides enough information on how the methodology can be replicated and used in a standardised way in future assessments. In my view, the methodology is described in a sufficient and adequate way to explain how authors processed their data. This is clear. But, at least to me, it is not clear how they used the data they generated to feed the evaluations following the IUCN guidelines and criteria.

In this sense, I am not sure that it is accurate to say that the methodology is a “key step of improving the protocol for Red List assessment in the effort to validate the taxonomic and spatial database”. If authors really want to keep this, they should make clear: a) to which ‘Red List assessment’ are they referring? To the IUCN ones? If so, please specify (and include references), and point out what exactly are the new things and benefits that the new methodology brings to the table; b) what are the differences, the improvements and advances of the new methodology with relation to the existing ones? c) how exactly the new approach was used when applying the IUCN criteria? d) how can the new methodology be replicated and implemented by other evaluators in other regions and countries?

In fact, if authors are confident about the potential of the new methodology (which I believe they are, and I myself would agree with them), I would suggest writing a second methodological paper, where they describe the methods in a didactic way (i.e. in a guideline format). I would specially recommend contacting the IUCN Amphibian Red List Authority to propose a joint paper incorporating the new methodology into the IUCN guidelines. But, to be honest, I don’t consider that the manuscript, as it is currently written, can be used as a model for future evaluations. Not because I don’t consider the methodology robust (which I do), but mainly because it is not described in a way that would allow its implementation by other evaluators. In other words, it is described as “this is how we did” but not as “this is how you should do”. I encourage authors to write a second methodological paper with the “how to do”.

Additional comments and suggestions can be found in the pdf file. Congratulations to the entire team on this great work! The overall quality of the work is impressive. I think now it is just a matter of improving the manuscript a little bit to reflect with more precision the high quality of all the work that was already done.
